# Comparative study on the gastrointestinal- and immune- regulation functions of Hedysari Radix Paeparata Cum Melle and Astragali Radix Praeparata cum Melle in rats with spleen-qi deficiency, based on fuzzy matter-element analysis

**DOI:** 10.1080/13880209.2022.2086990

**Published:** 2022-06-28

**Authors:** Yugui Zhang, Jiangtao Niu, Shujuan Zhang, Xinlei Si, Tian-Tian Bian, Hongwei Wu, Donghui Li, Yujing Sun, Jing Jia, Erdan Xin, Xingke Yan, Yuefeng Li

**Affiliations:** a College of Pharmacy, Gansu University of Chinese Medicine, Lanzhou, PR China; b Key Laboratory of Standard and Quality of Chinese Medicine Research of Gansu, Engineering Research Center of Chinese Medicine Pharmaceutical Process of Gansu, Gansu University of Chinese Medicine, Lanzhou, PR China; c College of Acupuncture-Moxibustion and Tuina, Laboratory of Molecular Biology, Gansu University of Chinese Medicine, Lanzhou, PR China; d Scientific Research and Experimental Center, Gansu University of Chinese Medicine, Lanzhou, PR China

**Keywords:** Traditional Chinese medicinal herb, tonifying middle and replenishing qi, the efficacy difference, gastrointestinal dysfunction, immune disorders, mathematical language

## Abstract

**Context:**

Hedysari Radix Praeparata Cum Melle (HRPCM) and Astragali Radix Praeparata Cum Melle (ARPCM) are used interchangeably in clinics to treat spleen-qi deficiency (SQD) symptom mainly including gastrointestinal dysfunction and decreased immunity, which has unknown differences in efficacy.

**Objective:**

To investigate the differences between HRPCM and ARPCM on intervening gastrointestinal- and immune-function with SQD syndrome.

**Materials and methods:**

After the SQD model was established, the Sprague–Dawley (SD) rats were randomly divided into nine groups (*n* = 10): normal; model; Bu-Zhong-Yi-Qi Pills; 18.9, 12.6 and 6.3 g/kg dose groups of HRPCM and ARPCM. Gastrointestinal function including d-xylose, gastrin, amylase vasoactive intestinal peptide, motilin, pepsin, H^+^/K^+^-ATPase, Na^+^/K^+^-ATPase, sodium-glucose cotransporter 1 (SGLT1), glucose transporter 2 (GLUT2) and immune function including spleen and thymus index, blood routine, interleukin (IL)-2, IL-6, interferon-γ (IFN-γ), tumour necrosis factor-α (TNF-α), immunoglobulin (Ig) M, IgA, IgG and delayed-type hypersensitivity (DTH) were detected. Finally, the efficacy differences were analysed comprehensively by the fuzzy matter-element method.

**Results:**

In regulating immune, the doses differences in efficacy between HRPCM and ARPCM showed in the high-dose (18.9 g/kg), but there were no differences in the middle- and low- dose (12.6 and 6.3 g/kg); the efficacy differences were primarily reflected in levels of IL-6, IFN-γ, TNF-α and IgM in serum, and the mRNA expression of IL-6 and IFN-γ in the spleen. In regulating gastrointestinal, the efficacy differences were primarily reflected in the levels of D-xylose, MTL, and GAS in serum, and the mRNA and protein expression of SGLT1 and GLUT2 in jejunum and ileum.

**Discussion and conclusions:**

HRPCM is more effective than ARPCM on regulating gastrointestinal function and immune function with SQD syndrome. Therefore, we propose that HRPCM should be mainly used to treat SQD syndrome in the future.

## Introduction

Hedysari Radix (HR), named ‘Hong-Qi’ in China, comes from the dry root of *Hedysarum polybotrys* Hand.-Mazz. Astragali Radix (AR), known as ‘Huang-Qi’ in China, is prepared from the dry root of *Astragalus membranaceus* (Fisch.) Bge. var. *mongholicus* (Bge.) Hsiao or *A. membranaceus* (Fisch.) Bge. Both of them belong to the Leguminosae family. Hedysari Radix Praeparata Cum Melle (HRPCM) and Astragali Radix Praeparata Cum Melle (ARPCM) are honey-processed products of HP and AR, respectively. HRPCM and ARPCM exhibit the effects of tonifying middle and replenishing qi. Thus, they are commonly used to treat spleen-qi deficiency (SQD) symptom such as fatigue, poor appetite, diarrhoea and weakened immunity (Editorial Committee of Chinese Pharmacopoeia 2020). However, HRPCM is esteemed an excellent medicine herbs to replace of ARPCM in the clinical setting from the North and South Dynasties period in China (Zhang et al. 2013; Peng et al. 2017). Until now, HRPCM is still used instead of ARPCM to treat SQD symptom in the northwest part (such as Gansu and Qinghai), as well as Hong Kong in China and some regions in southeast Asia (Zhang et al. 2013). Traditional Chinese medicine (TCM) theory holds that the effects of tonifying middle and replenishing qi are primarily relevant with tonifying spleen-qi (Wang et al. 2020). Furthermore, SQD impairs gastrointestinal function and reduces the ability to obtain nutrients, which then leads to the decline in immune function (Shang et al. 2013). Related studies in treatment of SQD symptom have also mainly focussed on the regulation of gastrointestinal function and immunoregulatory function (Tamura et al. 2004; Liu et al. 2015).

Our previous study showed that the high-performance liquid chromatography (HPLC) fingerprints of HRPCM and ARPCM were different. The HPLC fingerprint of HRPCM methanol extract (Li et al. 2019) has 19 common peaks but the HPLC fingerprint of ARPCM methanol extract has 8 common peaks (Zhang et al. 2021), which may explain why the two herbs differ in clinical use to regulate the gastrointestinal and immunoregulatory functions. However, no relevant pharmacological experiments can prove why HRPCM is more effective than ARPCM on tonifying middle and replenishing qi, especially on the regulation of gastrointestinal and immune function. Clinically, HRPCM and ARPCM cannot be accurately interchanged to use.

In this study, the efficacy difference between HRPCM and ARPCM on intervening gastrointestinal- and immune-regulation functions with SQD rats was compared. Fuzzy matter-element analysis is commonly used in the quantitative description of the relationships amongst the variables of concern that cannot be described by precise mathematical language (Wu et al. 2015). A correlation function is built to produce such a quantitative description (Wang et al. 2019). Here, this method was extended to eliminate the influences of various factors, and analysis models were established to compare comprehensively the efficacy difference between HRPCM and ARPCM on intervening gastrointestinal- and immunoregulation-regulation functions with SQD rats, respectively.

## Materials and methods

### Experimental herbs

HR (*H. polybotrys* Hand.-Mazz) and AR (*A. membranaceus* (Fisch.) Bge. var. mongholicus (Bge.) Hsiao samples were collected in May 2020 from Micang Mountain, Wudu District, Longnan City, Gansu Province, China. Rhubarb (*Rheum palmatum* L.) medicinal decoction pieces were purchased from Affiliated Hospital of Gansu University of Chinese Medicine. The three herbs were authenticated by Professor Mingwei Wang from the Department of Chinese Medicine Identification, School of Pharmacy, Gansu University of Chinese Medicine. Bu-Zhong-Yi-Qi Pills were purchased from Lanzhou Foci Pharmaceutical Co., Ltd. (Batch No. 19C38).

### Reagents

d-Xylose (Batch No. 20160302) was purchased from Tianjin Kaixin Chemical Industry Co., LTD. Phloroglucinol, glacial acetic acid, chloral hydrate, acetonitrile (chromatographically pure), methanoic acid (chromatographically pure), ammonium hydroxide, *n*-butyl alcohol, methyl alcohol (chromatographically pure) and acetone were all provided by Damao Chemical Reagent Factory (Tianjin, China; Batch Nos. 20190416, 20151123, 20180716, 20200616, 20200713, 20200702, 20200316, 20201225 and 20150501). Dinitrofluorobenzene (DNFB) was provided by Tokyo Chemical Industry Co. LTD (Batch No. VP2UE-RE). DNFB solution was prepared in 1% solution with 1:1 acetone-sesame oil solution before application.

### Preparation and quality analysis of HRPCM and ARPCM

The method of HRPCM was optimized by our group’s previous experiments (Niu et al. 2020). We collect a certain number of HR pieces, add refined honey infiltration for 1 h. Refined honey was diluted with distilled water (20% of honey). Approximately 25 g of refined honey was added to 100 g of HR pieces. The oven temperature was set to 70 °C, the baking time was set to 2.5 h and the HR tablets were laid to a thickness of 3 cm in a rectangular porcelain plate. After baking in the oven, the tablets were removed, cooled and set aside (Li et al. 2017; Niu et al. 2020). ARPCM was prepared by the same method as HRPCM to ensure the consistency of the experimental process and to avoid the influence caused by other factors. Subsequently, the general inspection items including moisture, total ash, extractum, total flavonoids and total polysaccharides have been determined according to the methods in references (Zhang 2021).

The chemical components were separated and identified by HPLC. The compositions of HRPCM were characterized using Agilent 1260 HPLC-DAD. The reference substances were vanillic acid, calycosin-7-*O*-β-D-glucoside, ononin, calycosin, and formononetin (all purity > 98%; batch numbers PS000459, PS000687, PS000671, PS010251, and PS000674, respectively. Push Bio-technology Co., Ltd., Chengdu, China). All reference substances were dissolved under ultrasonication in methanol and prepared as single-component reference solution with mass concentrations of 0.84, 0.62, 1.14, 1.41 and 1.43 mg/mL. The flavonoids and saponins of ARPCM were detected using Agilent 1260 HPLC-DAD and HPLC-ELSDA, respectively. Calycosin-7-*O*-β-D-glucoside, ononin, calycosin, formononetin, astragaloside I, astragaloside II, astragaloside III, and astragaloside IV were selected as the reference substances (purity > 98%; batch numbers PS000687, PS000671, PS010251, PS000674, PS000459, PS000462, PS200514-03, and PS010428, respectively; Push Bio-technology Co., Ltd., Chengdu, China). All reference substances were dissolved under ultrasonication in methanol and prepared as single-component reference solution with mass concentrations of 0.403, 0.325, 0.034, 0.104, 7.278, 1.022, 1.010 and 1.002 mg/mL. The specific HPLC analysis methods are listed in Online Supplementary Method 1.

### Animals and experimental protocols

**Figure 1.**
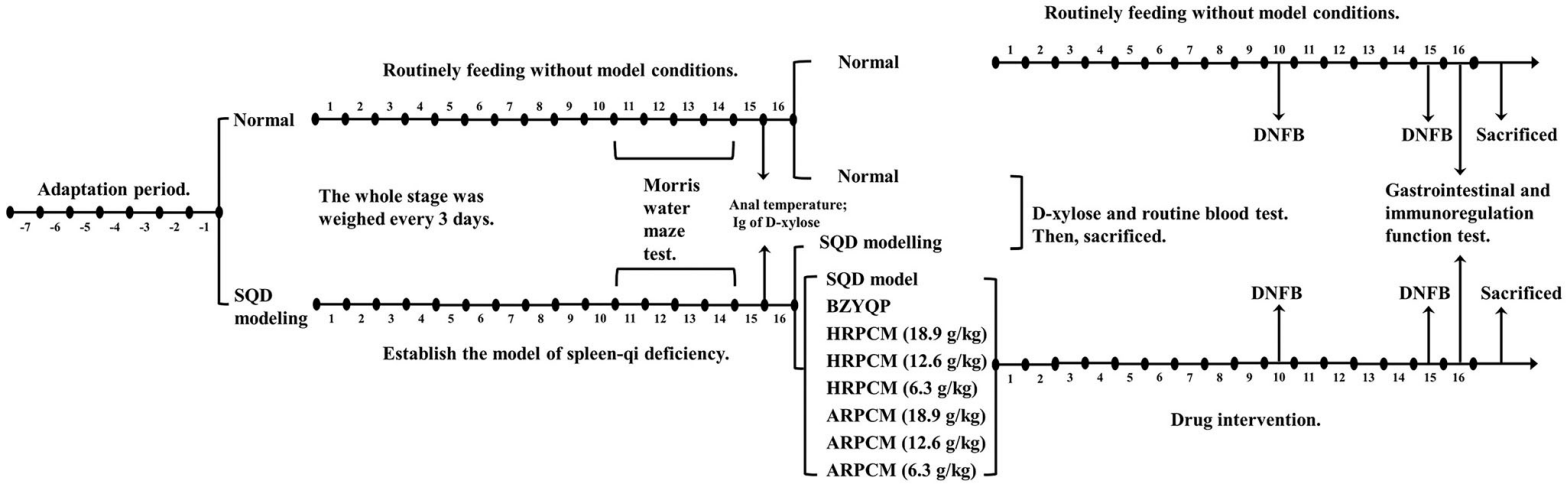
Overview of model procedure and drug-intervention procedure.

All animals and experimental protocols (Figure 1) were in accordance with the ethical standards of Gansu University of Chinese Medicine at which the studies were conducted (animal permit no. SCXK (Gan) 2015-0002). Approximately 6-week old, 110 male Sprague–Dawley (SD) rats, weighing 180 ± 20 g were obtained from specific pathogen free (SPF)-level laboratory of Gansu University of Chinese Medicine. Experimental rats were raised in the same SPF-level laboratory. The ambient temperature of the breeding room was controlled to 23 ± 2 °C, and its relative humidity was controlled to 45–50%.

After 7 d of adaptive feeding, 110 SD male rats were randomly divided into a normal group of 16 rats and a SQD modelling group (SQD modelling) of 94 rats. After successful establishment of the model, SQD modelling group were divided into a SQD model group (SQD model), Bu-Zhong-Yi-Qi Pill group (BZYQP, which is a kind of positive Chinese patent medicine with the effect of tonifying spleen qi), HRPCM low-dose group (6.3 g/kg), HRPCM medium-dose group (12.6 g/kg), HRPCM high-dose group (18.9 g/kg), ARPCM low-dose group (6.3 g/kg), ARPCM medium-dose group (12.6 g/kg) and ARPCM high-dose group (18.9 g/kg). Each group included 10 rats.

### Spleen-qi deficiency model and drug intervention

The SQD modelling rats used the three-factor composite modelling method of diet control, excessive fatigue, inducing diarrhoea (giving purgative Chinese herbal medicine) (Niu et al. 2020). However, they were fasted on odd-numbered days, given 75 g/kg/BW food on even-numbered days, and were intragastric administered (ig) with 1 g/mL rhubarb decoction of a dosage of 20 mL/kg at 12:00 every day. Every afternoon, the modelling rats were placed in a swimming tank with a water temperature of 25 ± 1 °C and water depth of 50 cm to swim. Meanwhile, a fuse weighing 10% of the body mass was attached onto the base of the rat-tail until they were exhausted (Zeng et al. 2019; Niu et al. 2020). After exhaustion, the rats were collected and dried. Modelling was performed for 15 d. The normal rats were routinely fed without model conditions.

The ig dosage for rats was calculated accurately according to the body-surface-area method. After the model was successfully established, rats in positive-control group were given 18.9 g/kg of BZYQP aqueous solution. Rats in the high-, medium- and low-dose groups of HRPCM and ARPCM were given 18.9, 12.6 and 6.3 g/kg, respectively, of HRPCM or ARPCM aqueous extract. Rats in the normal and SQD model were given equal doses of distilled water. The corresponding dose was administered 10 mL/kg every day at 10:00 am by ig and once a day lasting for 15 d. Except for the normal, the remaining groups continued to apply the modelling conditions. On the 10th day of drugs intervention, each rat was depilated on the abdomen, and the size was just 3 cm × 3 cm. The depilated abdomen was sensitized with a 50 μL DNFB solution. Five days after sensitization, 10 μL of DNFB solution was evenly wiped onto the right ear of each rat for an attack for delayed-type hypersensitivity (DTH).

### Behaviour experiments, d-xylose test and blood routine test in modelling process

The bodyweight changes of rats in each group were observed during the model establishment and drugs intervention. The rats were weighed every 3 d. Meanwhile, the physical signs of changes were observed, such as coat colour, tail colour, arching back, bunching, movement, diet, stool colour and shape, squinting (Zhang et al. 2020). Rectal temperature changes of rats in each group were also detected after the end of the modelling model and medicine intervention, respectively. Moreover, the Morris water maze test was primarily used to examine the learning and memory abilities of different subgroups of rats (Wu et al. 2020). The tests contained the oriented-navigation test and the spatial probe test (Gong et al. 2020). Escape latency in the oriented-navigation test and the number of times crossing the platform area, target quadrant retention time and percentage in the spatial probe test were recorded. The SQD modelling rats were fasted overnight on the 15th day of modelling. Six rats were randomly selected from the normal and SQD modelling respectively on the 16th morning. Then, d-xylose and routine blood including RBC, HGB, WBC, LYM and PLT were analysed. The specific test methods are listed in Online Supplementary Method 2. The success of establishing the SQD model was determined by combining the results of the above indicators and behaviour experiments (Zeng et al. 2019; Huang et al. 2020).

### Sample preparation

All rats were fasted overnight on the 15th day of drug intervention, and then 5% d-xylose solution with a dose of 10 mL/kg was given by ig on the 16th morning. Rats were anesthetized after 1 h. Then, 1 mL of blood sample was collected directly for blood routine tests, including WBC, LYM, HGB and RBC. About 5–8 mL of blood sample was again collected into non-anticoagulated vacuum blood-collection tubes and stored in a refrigerator at 4 °C for 1 h. The samples were then centrifuged at 3000 rpm for 15 min at 4 °C, after which the liquid supernatant was placed in 2 mL EDTA tubes and stored in the refrigerator at −80 °C for further analysis.

Rats were sacrificed after blood collection. The ears, spleen, thymus, stomach and small intestine of rats were immediately removed. After washing blood stains, fascia and fatty tissue were removed. Spleen and thymus were accurately weighed for the determination of thymus index and spleen index. The ears of all rats were removed and 8 mm diameter holes were punched through. The difference in mass between the right and left ear pieces was used to evaluate DTH. After weighing, the spleen was divided equally into two halves, one half was fixed with 4% paraformaldehyde fixative and used for observation of HE-stained pathological sections, and the other half was stored in the refrigerator at −80 °C to determine the expression levels of interleukin (IL)-6 mRNA and interferon-γ (IFN-γ) mRNA. The content of the small intestine was washed off carefully, and the duodenum, jejunum and ileum were preserved. The duodenum was prepared for hematoxylin–eosin (HE) staining observation. The jejunum was equally divided into two segments that are the proximal duodenum (J-Pd) and the distal duodenum (J-Dd). The J-Pd and the ileum were used to determine the protein and mRNA expression levels of SGLT1 and GLUT2, and the J-Dd was used for the preparation of jejunal homogenate and HE-staining observation. The J-Dd and stomach were gently cut into pieces with forceps, collected into sterile centrifugal tube, and added 9 times the amount of PBS solution with pH 7.2–7.4. After being fully homogenized, and then centrifuged at 3000 rpm for 20 min, the liquid supernatant was placed in 2 mL EDTA tubes. The tissue homogenate of stomach and J-Dd were stored in the refrigerator at −80 °C for further analysis. The above method of tissue homogenate preparation was primarily from the enzyme-linked immunosorbent assay (ELISA) kit.

### Measurements of biochemical indicators

The serum samples were assayed for d-Xylose, MTL, GAS, AMS, VIP, IL-2, IL-6, IFN-γ, tumour necrosis factor-α (TNF-α), immunoglobulin (Ig)A, IgG and IgM. The samples of gastric tissue homogenization were assayed for H^+^/K^+^-ATPase and pepsin. The samples of J-Dd tissue homogenization were assayed for Na^+^/K^+^-ATPase. Rat d-Xylose ELISA kit was provided by Enzyme-linked Biotechnology Co., Ltd, Shanghai, China (Batch No. ml015649). Rat MTL, GAS, AMS, VIP, IL-2, IL-6, IFN-γ, TNF-α, IgA, IgG, IgM, Pepsin, H^+^/K^+^-ATPase and Na^+^/K^+^-ATPase ELISA kits were purchased from Feiya Biological Technology Co., Ltd, Jiangsu, China. (Batch Nos. MM-0491R1, MM-20284R1, MM-21167R, MM-0445R1, MM-0192R1, MM-0190R1, MM-0198R1, MM-0180R1, MM-0062R1, MM-0064R1, MM-0065R1, MM-0288R1, MM-20257R1 and MM-43903R1, respectively). All indicators were assayed with ELISA kits according to the supplier’s instruction manuals.

### Histopathological evaluation

After fixing with 4% paraformaldehyde fixative for 1 week, the spleen, stomach, duodenum, jejunum and ileum were embedded in paraffin, sliced, baked, stained with HE and observed under an inverted microscope at 200×.

### Quantitative RT-PCR analysis

Total RNA was extracted from spleen, the J-Pd and the ileum tissues with TRIeasy™ Total RNA Extraction Reagent (10606ES60, YEASEN Biotech Co. Ltd, Shanghai, China). The first-strand complementary DNA (cDNA) was synthesized using ‘Hifair® III 1st Strand cDNA Synthesis SuperMix for qPCR (gDNA digester plus)’ (11141ES10, YEASEN Biotech Co. Ltd, Shanghai, China). Quantitative real-time qPCR (qRT-PCR) analysis was performed on a real-time PCR system according to ‘Hieff UNICON® Power qPCR SYBR Green Master Mix (Antibody technique, Low Rox)’ (11202ES08, YEASEN Biotech Co. Ltd, Shanghai, China). mRNA content was calculated by 2^−ΔΔCt^ relative quantification with β-actin as an internal reference. All mRNA primer sequences were shown in Table 1. All primers were designed and synthesized by Accurate Biology Co., Ltd, Hunan, China.

**Table 1.** Primers sequences used for qRT PCR.

Gene	Forward primer	Reverse primer
SGLT1 (Slc5a1)	5′-TCTGCCACGCCTATTTTGC-3′	5′-GCCAGTTTCCCCTTACCACTAC-3′
GLUT2 (Slc2a2)	5′-CAGCACATACGACACCAGACG-3′	5′-AGAACGAGGCGACCATTCC-3′
IL-6	5′-GACAGCCACTGCCTTCCCTA-3′	5′-GAATTGCCATTGCACAACTCTT-3′
IFN-γ	5′-CAACCCACAGATCCAGCACA-3′	5′-TCAGCACCGACTCCTTTTCC-3′
β-actin	5′-GGAGATTACTGCCCTGGCTCCTA-3′	5′-GACTCATCGTACTCCTGCTTGCTG-3′

### Western blot

Total protein from the J-Pd and the ileum tissues were extracted, separated by SDS-PAGE and transferred to a polyvinylidene fluoride (PVDF) membrane. The PVDF membrane was blocked with 5.0% skim milk powder, placed in the protein primary antibody diluent GLUT2 (1:500 dilutions, 20436-1-AP, Proteintech Group, Inc., Rosemont, IL, USA) and SGLT1 (1:800 dilutions, ab14686, Abcam, Cambridge, UK) overnight at 4**°**C. After washing with TBST, the membrane was incubated with the secondary antibody for 1 h at room temperature. The internal control protein was β-actin. The blot was visualized using the chemiluminescence imaging system (MiniChemi 610, LIUYI Biotechnology Co. Ltd., Beijing, China.). Protein expressions were quantified through the Image J software and normalized against β-actin and the administration group.

### The fuzzy matter-element analysis

#### Fuzzy matter-element concept

In fuzzy matter-element analysis, the fuzzy matter-element *R* comprises *M*, *C* and *X*. *M* is a variable or matter, *C* is the characteristics of the matter and *X* is the value of the matter. Thus, *R* is also defined as *R* = (*M*, *C*, *X*). The matter, characteristics of the matter, and value of the matter are called matter-element three elements. If the X is a fuzzy set in the matter-element model, then *R* represents the fuzzy matter element (Han et al. 2019). In general, n-dimensional matter elements of m things are combined to form n-dimensional compound fuzzy matter elements of m things (namely, *R*_mn._), which can be recorded as:

(1) $$R_{mn}=[\begin{array}{ccccc} & M_{1} & M_{2} & \cdots & M_{m}\\ C_{1} & X_{11} & X_{21} & \cdots & X_{m1}\\ C_{2} & X_{12} & X_{22} & \cdot \cdot \cdot & X_{m2}\\ \vdots & \vdots & \vdots & \cdot \cdot \cdot & \vdots \\ C_{n} & X_{n2} & X_{n3} & \cdot \cdot \cdot & X_{mn} \end{array}]$$

where *X_ij_* (*i*= 1, 2, …, *n*; *j*= 1, 2, …, *m*) is the *j*th matter element fuzzy value of *i*th matter. In this study, *C* is gastrointestinal- and immune-function-related indicators, *M* is the different groups and *X* is the corresponding value of each indicator.

#### Determination of subordination membership to fuzzy matter-element

The subordination membership degree indicates the fuzzy value corresponding to the single-effect parameter of the gastrointestinal- and immune- function-related indicators belonging to the standard value of each single-effect parameter (Li et al. 2020).

In Equation (1), the membership degree *X_ij_* calculated by the subordinate membership principle is used to replace *Ų_ij_* in Equation(2). The subordinate membership fuzzy matter-element *R’*mn is formed, namely:

(2) $${R’}_{mn}=[\begin{array}{ccccc} & M_{1} & M_{2} & \cdots & M_{m}\\ C_{1} & {Ų}_{11} & {Ų}_{21} & \cdots & {Ų}_{m1}\\ C_{2} & {Ų}_{12} & {Ų}_{22} & \cdot \cdot \cdot & {Ų}_{m2}\\ \vdots & \vdots & \vdots & \cdot \cdot \cdot & \vdots \\ C_{n} & {Ų}_{n2} & {Ų}_{n3} & \cdot \cdot \cdot & {Ų}_{mn} \end{array}]$$

where *R*’*mn* is the priority membership fuzzy matter-element; *Ųij* (*i*= 1, 2, …, n; *j*= 1, 2, …, *m*) is the fuzzy value of the *j*th matter element of *i*th matter. However, as far as the overall scheme evaluation is concerned, the value and the trend of each indicator are different. Some indicators are larger and more optimal, but some are smaller and more optimal. Therefore, different calculation equations are used for different membership degrees (Xia et al. 2018). It can be recorded as:

The larger is the more optimal:

(3) $${Ų}_{ij}=\frac{{\;X}_{ij}\;}{{\;\mathrm{MaxX}}_{ij}}$$

The smaller is the more optimal:

(4) $${Ų}_{ij}=\frac{{\;\mathrm{MinX}}_{ij}\;}{X_{ij}}$$

where Max*X_ij_* is the maximum value of these indicators in each group; Min*X_ij_* is the minimum value of these indicators in each group.

#### Determination of standard fuzzy matter-element and simple absolute-value compound fuzzy matter-element

The n-dimensional standard fuzzy matter-element *R′*_0n_ is the maximum or minimum value of the corresponding indicator in each group in the fuzzy matter-of-priority membership (Li et al. 2020). The equation is shown in (5).

(5) $${R'}_{0n}=[\begin{array}{cc} & M_{0}\\ C_{1} & {Ų}_{01}\\ C_{2} & {Ų}_{02}\\ \vdots & \vdots \\ C_{n} & {Ų}_{0n} \end{array}]$$

where *M*_0_ represents the standard sample, and *Ų*_0j_ (*j*= 1, 2, …, *m*) is the maximum or minimum value of the *j*th indicators. If △*ij* (*i*= 1, 2…*n*；*j*= 1, 2…*m*) represents the absolute value of each difference value between *R′*_0n_ and *R′*_mn_. Then a simple difference absolute-value compound fuzzy matter element R_△_ is formed. The equation is shown in (6).

(6) $$R_{\Delta }=[\begin{array}{ccccc} & M_{1} & M_{2} & \cdots & M_{m}\\ C_{1} & \Delta_{11} & \Delta_{21} & \cdots & \Delta_{m1}\\ C_{2} & \Delta_{12} & \Delta_{22} & \cdot \cdot \cdot & \Delta_{m2}\\ \vdots & \vdots & \vdots & \cdot \cdot \cdot & \vdots \\ C_{n} & \Delta_{n2} & \Delta_{n3} & \cdot \cdot \cdot & \Delta_{mn} \end{array}]$$

where

(7) $$\Delta_{ij}=|{Ų}_{0i}-{Ų}_{ji}|$$

#### Determination of weighting for evaluation index

The coefficient of variation method was used to calculate the quality evaluation indicators’ weights of TCM (Chen and Deng 2014; Zhou et al. 2016). The calculation steps are as follows.

The mean value ${\bar{\text{X}}}_{\text{i}}$ of the ith evaluation indicators are calculated, namely:

(8) X¯i=1n∑i=1nXi

The mean square error ${\bar{\text{D}}}_{\text{i}}$ of the ith evaluation indicators are calculated, namely:

(9) D¯i=[1n∑i=1n(xi−x¯i)2]12

The coefficient of variation δ*i* of the ith evaluation indicators are calculated, namely:

(10) $$\delta_{i}={\bar{\text{D}}}_{i}/{\bar{\text{x}}}_{\text{i}}$$

The weight Wi of the ith evaluation indicators is calculated, namely:

(11) $$W_{i}=\delta_{i}{\left(\sum_{i=1}^{n}\delta_{i}\right)}^{-1}$$

#### Closeness and comprehensive evaluation

Closeness reflects the close degree between the sample to be evaluated and the standard sample. The larger the value, the closer it was, and vice versa. Therefore, the evaluation samples can be sorted or classified according to the closeness (Xia et al. 2018). In this study, the Euclidean closeness ρHj (*j* = 1, 2……*m*) is used as the evaluation standard to calculate and construct the closeness compound fuzzy matter element Rρ_H_, namely:

(12) $$R_{\text{ρH}}=[\begin{array}{ccccc} & M_{1} & M_{2} & \cdots & M_{m}\\ {\text{ρH}}_{j} & {\text{ρH}}_{1} & {\text{ρH}}_{2} & \cdots & {\text{ρH}}_{m} \end{array}]$$

Where ρHj=1−[Σi=1nWi×Δij]12

### Statistical methods

All data were processed with SPSS version 19.0 (SPSS, Inc., Chicago, IL) and Paragraph Prism version 8.0 (GraphPad Software, La Jolla, CA) software. Data from all the experiments were expressed as mean ± standard error (SE). The statistical significance of the results between each treated group was analysed by one-way analysis of variance (ANOVA), and the post-test was Dunnett’s multiple comparisons test. *p*< 0.05 was considered statistically different.

## Results

### Qualitative and quantitative analysis of HRPCM and ARPCM

**Figure 2.**
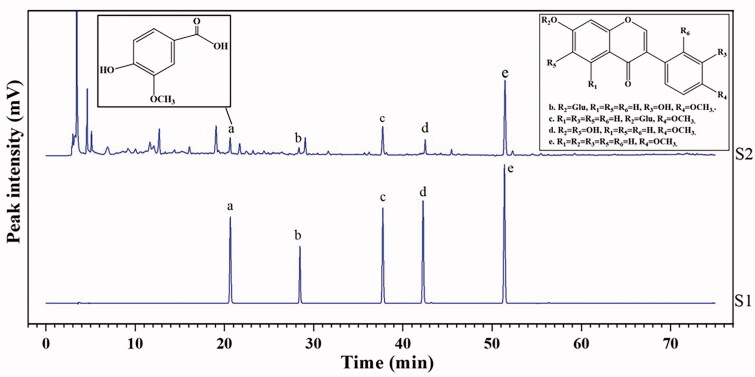
HPLC analysis of HRPCM. S1, Mixed reference. (a) Vanillic acid; (b) calycosin-7-*O*-β-d-glucoside; (c) ononin; (d) calycosin; (e) formononetin; S2, HPPCM.

**Figure 3.**
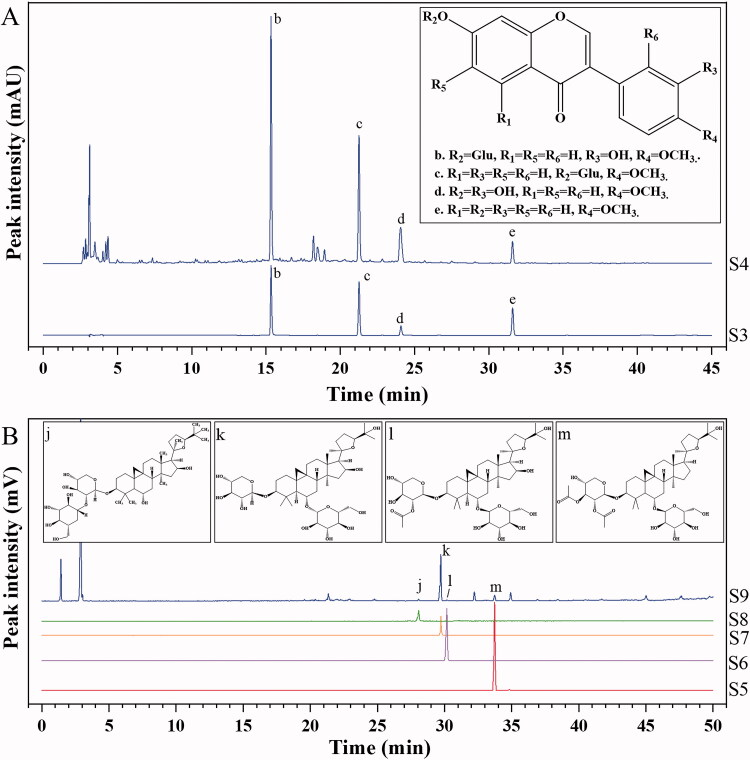
HPLC analysis of ARPCM. (A) HPLC-DAD chromatogram. (B) HPLC-ELSDA chromatogram. (b) calycosin-7-*O*-β-d-glucoside; (c) ononin; (d) calycosin; (e) formononetin; (j) astragaloside III; (k) astragaloside IV; (l) astragaloside II; (m) astragalosideI. (Note: S9. ARPCM. Mixed reference in B is prone to miscellaneous peaks, so single reference materials were selected, respectively).

Results of the general inspection items and indicative components of HPLC analysis in HRPCM and ARPCM are shown in Table 2. We assayed the contents of total polysaccharides and total flavonoids in HRPCM were higher than ARPCM in this batch. This result may be one of the reasons for the difference in the overall efficacy between HRPCM and ARPCM. Previous studies have also proven that total polysaccharides and total flavonoids in HRPCM and ARPCM are related to immune regulation and absorption metabolism, respectively (Jiao et al. 2000; Hu et al. 2010; Luo et al. 2021). The HPLC analysis profiles of HRPCM (Figure 2) and ARPCM (Figure 3) were also established based on a previous study to identify the indicative component and calculate their contents (Zhang 2021). We found that the chromatographic peaks between HRPCM and ARPCM were significantly differed. Vanillic acid was identified in HRPCM but was not in ARPCM. Saponins such as astragalosideI, astragaloside II, astragaloside III and astragaloside IV were identified in ARPCM but not in HRPCM. These results were consistent with previous studies (Ma et al. 2002), which demonstrated that the content of saponins is not easily detected in HRPCM. These results were in line with the requirements of Chinese Pharmacopoeia, indicating that two drugs were successfully qualified, thereby providing basis for the follow-up efficacy experimental study of HRPCM and ARPCM.

**Table 2.** Results of the general inspection items and HPLC analysis (*n* = 3, $\overset{\;}{\text{mean}}\pm \;\text{SE}$).

Items	Inspection items	HRPCM	ARPCM
General inspection	Moisture (%)	7.41 ± 0.07	7.70 ± 0.05 **↑**
	Total ash (%)	3.59 ± 0.22	2.98 ± 0.06 **↓**
	Extractum (%)	40.21 ± 0.78	35.57 ± 0.54 **↓**
	Total flavonoids (%)	10.03 ± 0.17	8.96 ± 0.64 **↓**
	Total polysaccharides (%)	14.75 ± 0.39	11.01 ± 0.55 **↓**
HPLC analysis	Vanillic acid (mg/g)	0.032 ± 0.002	–
	Calycosin-7-*O*-β-D-glucoside (mg/g)	0.008 ± 0.002	0.245 ± 0.002 **↑**
	Ononin (mg/g)	0.196 ± 0.007	0.146 ± 0.001 **↓**
	Calycosin (mg/g)	0.046 ± 0.003	0.026 ± 0.002 **↓**
	Formononetin (mg/g)	0.393 ± 0.004	0.009 ± 0.001 **↓**
	Astragaloside I (mg/g)	–	0.097 ± 0.001
	Astragaloside II (mg/g)	–	0.079 ± 0.001
	Astragaloside III (mg/g)	–	0.177 ± 0.005
	Astragaloside IV (mg/g)	–	1.177 ± 0.006

### Evaluation of SQD model

**Figure 4.**
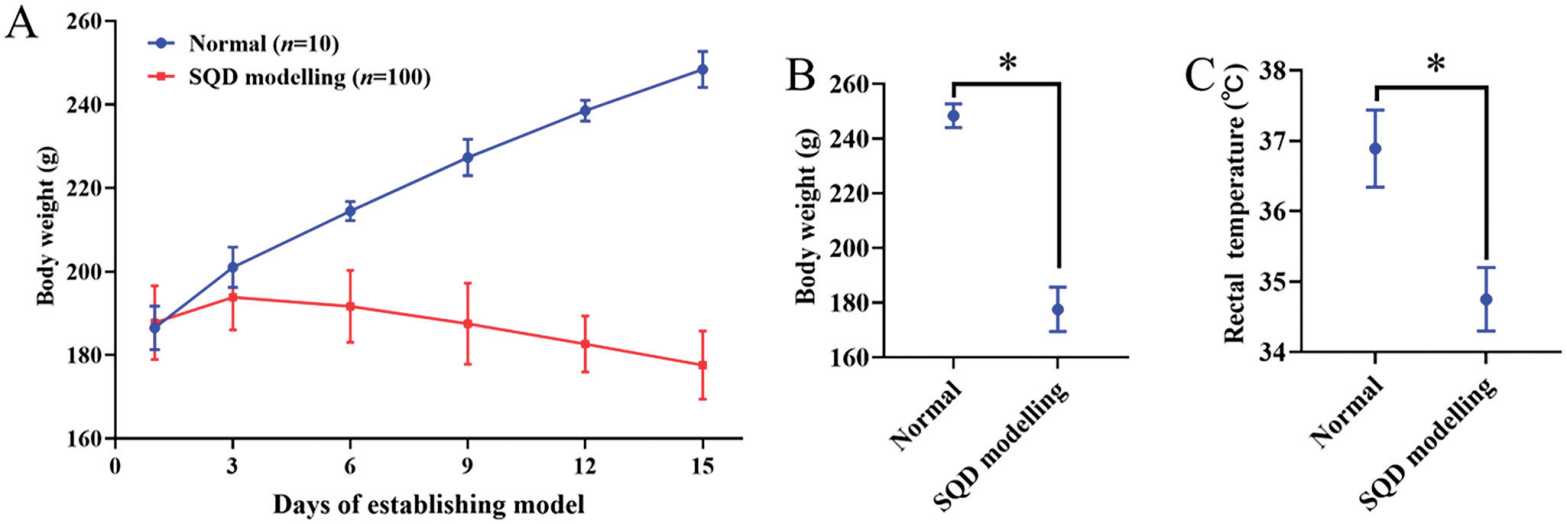
The weight change and rectal temperature during modelling. (A) Weight change every three days during modelling. (B) Body weight between normal and SQD modelling on 15th of establishing model (Normal, *n*= 16; SQD modelling, *n*= 94). (C) Rectal temperature between normal and SQD modelling on 15th of establishing model (Normal, *n*= 16; SQD modelling, *n*= 94). All data were presented as the mean ± SE and were analysed by ANOVA followed by Dunnett’s multiple comparison tests. *p< 0.05 when compared with normal. SQD modelling: spleen-qi deficiency modelling group.

**Figure 5.**
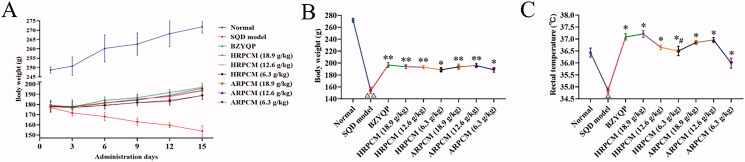
The weight change and rectal temperature during drug intervention. (A) Weight change every 3 d. (B) Body weight 15 d after drug administration. (C) Rectal temperature 15 d after drug administration. All data were presented as the mean ± SE (*n*= 8.) and were analysed by ANOVA followed by Dunnett’s multiple comparison tests. ^△^*p* < 0.05 and ^△△^*p* < 0.01 when compared with normal rats. *p < 0.05 and **p < 0.01 when compared with SQD model. ^#^*p* < 0.05 and ^##^*p* < 0.05 when compared with ARPCM of corresponding doses. Thus, HRPCM (18.9 g/kg) compared with ARPCM (18.9 g/kg); HRPCM (12.6 g/kg) compared with ARPCM (12.6 g/kg); HRPCM (6.3 g/kg) compared with ARPCM (6.3 g/kg). SQD modelling, spleen-qi deficiency modelling group. BZYQP: Bu-Zhong-Yi-Qi-Pill.

**Figure 6.**
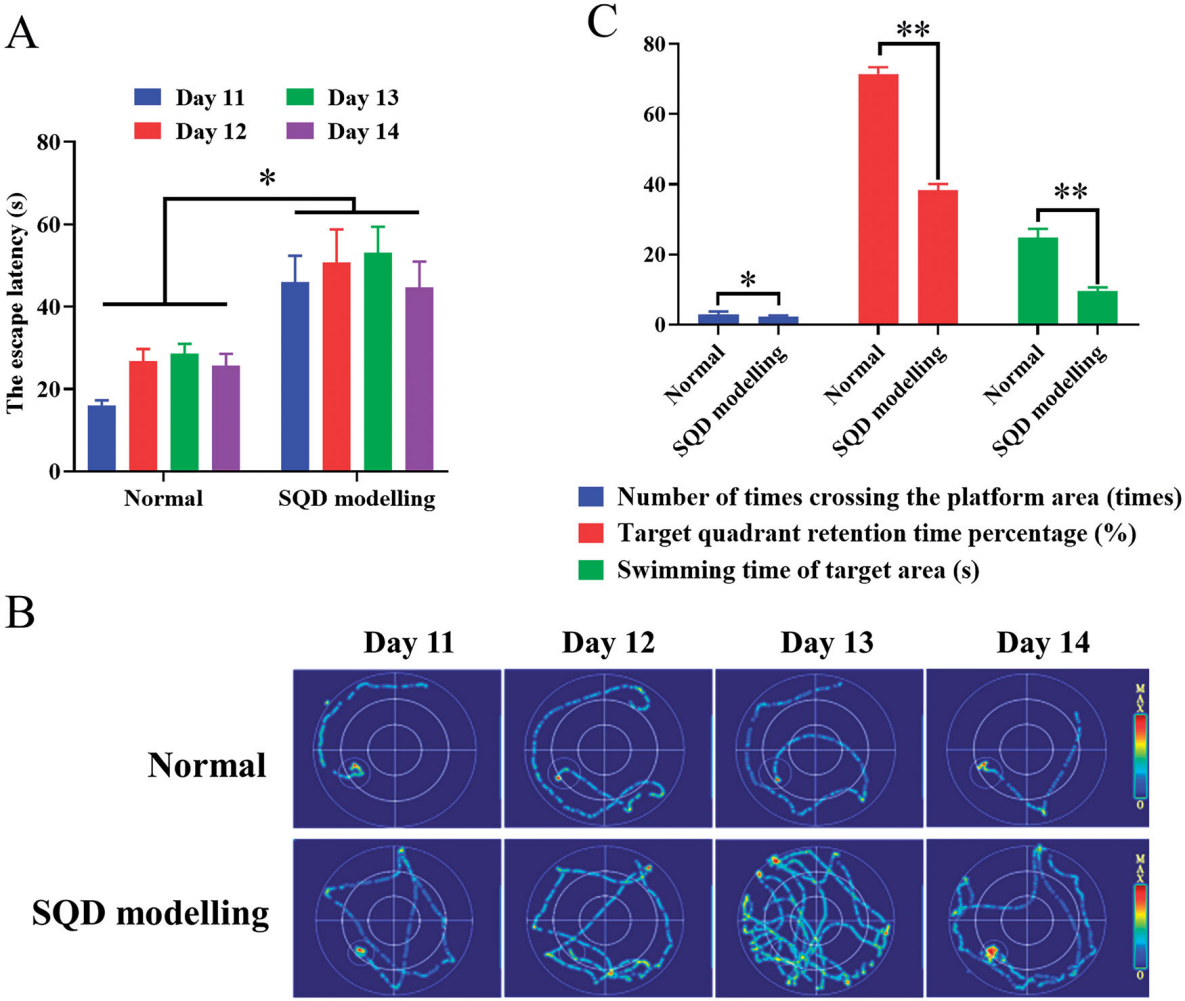
Results of the Morris water maze experiment. (A) Escape latency in the oriented-navigation experiment (Normal, *n*= 10; SQD modelling, *n*= 40.). (B) Swimming trajectory graph in the oriented-navigation experiment. (C) Results of spatial-probe experiment, including the number of times crossing the platform area, target quadrant retention time percentage, and swimming time of target area within 120 s (Normal, *n*= 10; SQD modelling, *n*= 40.). All data were presented as the mean ± SE and were analysed by ANOVA followed by Dunnett’s multiple comparison tests. *p< 0.05 and **p< 0.01 when compared with normal. SQD modelling: spleen-qi deficiency modelling group.

**Figure 7.**
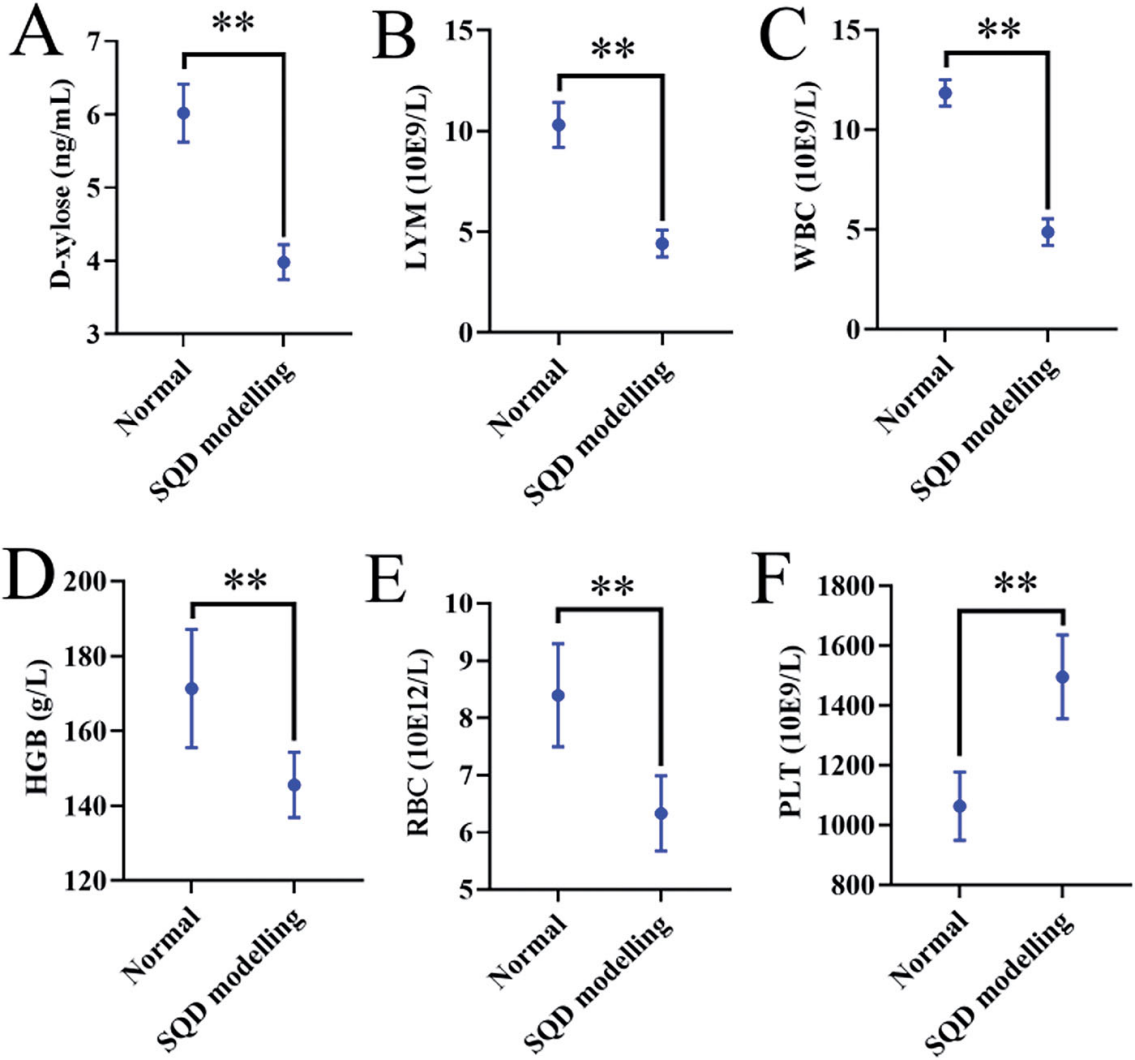
Results of D-xylose absorption and blood routine test. (A) D-xylose absorption and blood routine tests after 15 d of establishing model. (B–F) The blood routine test included LYM, WBC, HGB, RBC, and PLT when evaluating the model of SQD. All data were presented as the mean ± SE and were analysed by ANOVA followed by Dunnett’s multiple comparison tests. * *p* < 0.05 and **p < .0.01 when compared with normal. SQD modelling: spleen-qi deficiency modelling group; LYM: lymphocyte; WBC: white blood cell; HGB: haemoglobin; RBC: red blood cell; PLT: phosphate buffer solution.

The rats were healthy without abnormal signs before the establishing model. After establishing the model, the SQD modelling rats showed significant loose stools, serious squinting, sleepiness with tiredness and drowsiness, sparse and lossy fur, sluggish reflexes with lethargy compared with the normal rats. All of the above signs were similar to the symptoms of TCM SQD symptoms pattern according to the reference (Zhang et al. 2017, 2020). The body weight and rectal temperature of SQD modelling rats were significantly downregulated compared with normal rats (Figure 4). During drug intervention, the body weight of each administration and normal groups gradually increased, but the body weight of SQD model continuously decreased as model establishment continued (Figure 5(A)). Compared with the corresponding dose groups between HRPCM and ARPCM, there were only significant difference between HRPCM (6.3 g/kg) and ARPCM (6.3 g/kg) in rectal temperature (*p* < 0.05, Figure 5(C)). The remaining corresponding doses were not statistically significant not only in rectal temperature, but also in body weight. These results indicated that the interventions of all administration groups contributed to restoring the body weight, and significantly upregulating rectal temperature. In morris water maze test, the escape latency of the SQD modelling rats was significantly longer than the normal rats in general (*p* < 0.05; Figure 6(A)). The swimming trajectory graph is shown in Figure 6(B). The times of crossing the platform area, target-quadrant retention time percentage, and swimming time of target area all of SQD modelling rats were significantly shorter than those of normal rats (*p* < 0.05, *p* < 0.01 and *p* < 0.05; Figure 6(C)). Fifteen days after establishing the model, it was found that lower levels of d-xylose in the SQD modelling rats than in the normal rats (*p* < 0.01; Figure 7(A)). The results of blood routine indices such as LYM, WBC, HGB and RBC showed lower levels in the SQD modelling rats than that in the normal rats (*p* < 0.01; Figure 7(B–E)). PLT level showed the reverse trend and was higher in SQD modelling rats than that in normal rats (*p* < 0.01; Figure 7(F)). Overall, these results directly indicated that the SQD model was established successfully.

### Differences of the pharmacological effects on regulating gastrointestinal functions

#### Differences in biochemical index detection

**Figure 8.**
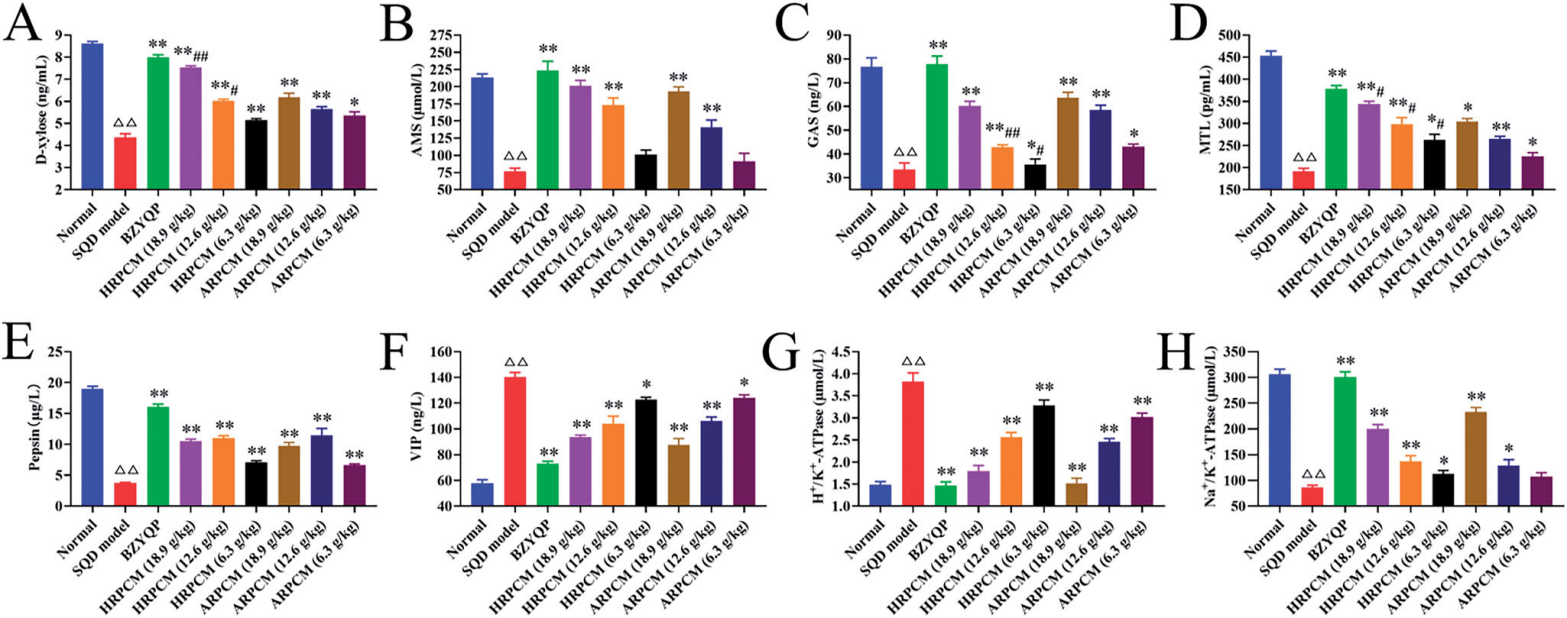
Results of gastrointestinal-function-related indices in each group of rats after 15 d drug administration. (A) D-xylose (B) AMS (C) GAS. (D) MTL (E) Pepsin (F) VIP. (G) H+/K+-ATPase (H) Na+/K+-ATPase. All data were presented as the mean ± SE (*n*= 8) and were analysed by ANOVA followed by Dunnett’s multiple comparison tests. ^△^*p* < 0.05 and ^△△^*p* < 0.01 when compared with normal rats. *p < 0.05 and **p < 0.01 when compared with SQD model. ^#^*p* < 0.05 and ^##^*p* < 0.05 when compared with ARPCM of corresponding doses. Thus, HRPCM (18.9 g/kg) compared with ARPCM (18.9 g/kg); HRPCM (12.6 g/kg) compared with ARPCM (12.6 g/kg); HRPCM (6.3 g/kg) compared with ARPCM (6.3 g/kg). AMS: amylase; GAS: gastrin; MTL: motilin; VIP: vasoactive intestinal peptide.

**Figure 9.**
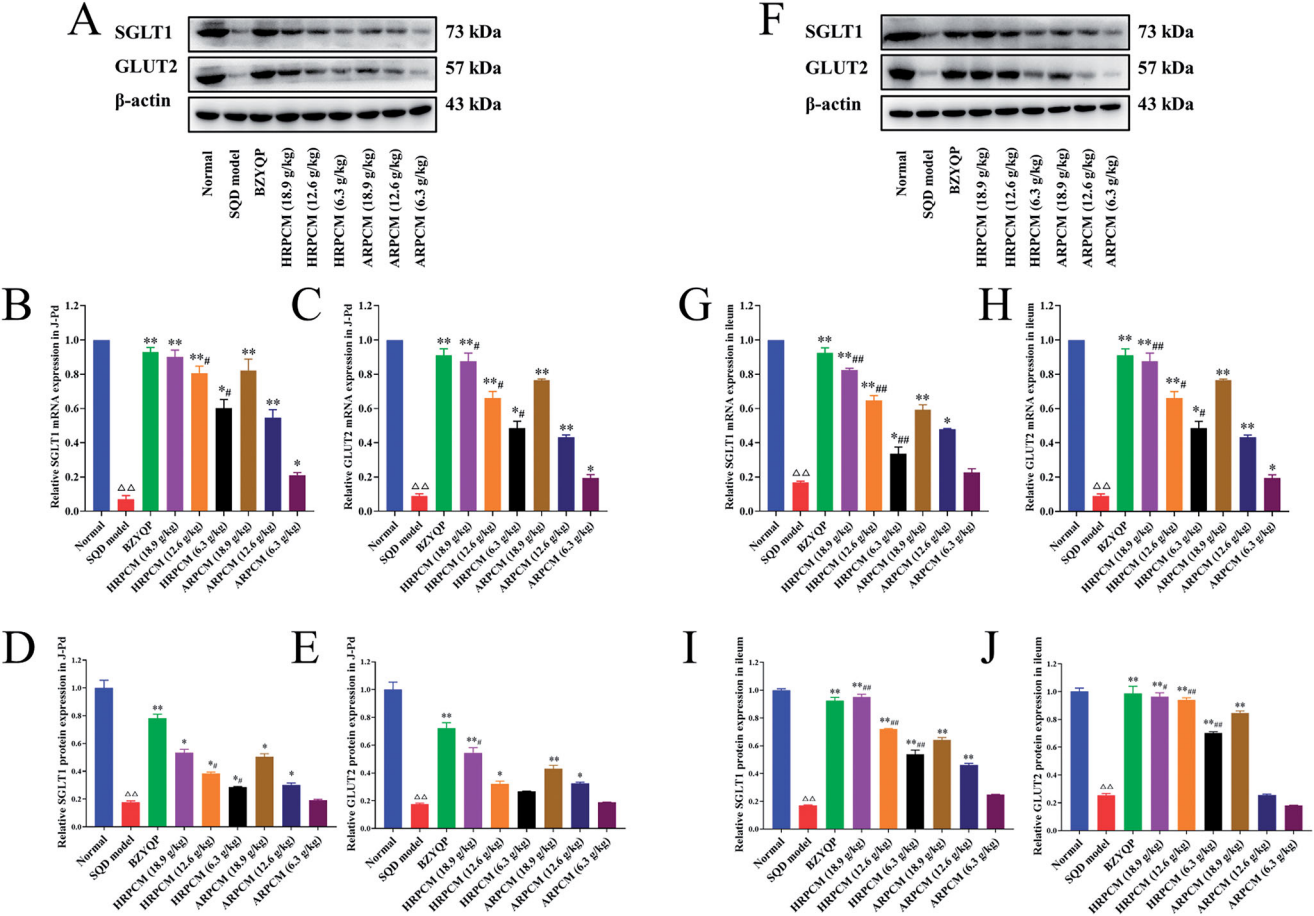
Related protein and gene expression in the J-pd and ileum. (A) Western blotting in the J-pd. (B) Relative SGLT1 mRNA expression in the J-pd. (C) Relative SGLT1 protein expression in the J-pd. (D) Relative GLUT2 mRNA expression in the J-pd. (D) Relative GLUT2 protein expression in the J-pd. (F) Western blotting in the ileum. (G) Relative SGLT1 mRNA expression in the ileum. (H) Relative SGLT1 protein expression in the ileum. (I) Relative GLUT2 mRNA expression in the ileum. (J) Relative GLUT2 protein expression in the ileum. (K) Pathological observations of stomach, duodenum, jejunum and the ileum (200×). All data were presented as the mean ± SE (*n*= 3) and were analysed by ANOVA followed by Dunnett’s multiple comparison tests. ^△^*p* < 0.05 and ^△△^*p* < 0.01 when compared with normal rats. *p < 0.05 and **= < 0.01 when compared with SQD model. ^#^*p* < 0.05 and ^##^*p* < 0.05 when compared with HPAR of corresponding doses. In other words HRPCM (18.9 g/kg) compared with ARPCM (18.9 g/kg); HRPCM (12.6 g/kg)compared with ARPCM (12.6 g/kg); HRPCM (6.3 g/kg) compared with ARPCM (6.3 g/kg). J-Pd, the jejunum of proximal duodenum.

Fifteen days after drug intervention, compared with the corresponding dose groups between HRPCM and ARPCM, respectively, the levels of AMS, pepsin, VIP, Na^+^/k^+^-ATPase, and H^+^/k^+^-ATPase were not statistically significant difference (*p* > 0.05; Figure 8(B,E,F,G,H)). The D-xylose levels were significantly higher in HRPCM (18.9 and 12.6 g/kg) than that in ARPCM (18.9 and 12.6 g/kg), respectively (*p* < 0.01; Figure 9(A)), but no statistical significance existed between HRPCM (6.3 g/kg) and ARPCM (6.3 g/kg) (*p* > 0.05; Figure 9(A)). The GAS levels were significantly lower in HRPCM (12.6 and 18.9 g/kg) than in ARPCM (12.6 and 18.9 g/kg), respectively (*p* < 0.01, *p* < 0.05; Figure 9(C)), but no statistical significance existed between HRPCM (18.9 g/kg) and ARPCM (18.9 g/kg) (*p* > 0.05; Figure 9(C)). The MTL levels were significantly higher in HRPCM (18.9, 12.6 and 6.3 g/kg) than in ARPCM (18.9, 12.6 and 6.3 g/kg), respectively (*p* < 0.05; Figure 9(D)). These results showed that differences existed in regulating the levels of MTL, GAS and D-xylose, but no significant effect was found in regulating the levels of Pepsin, AMS, VIP, Na^+^/K^+^-ATPase and H^+^/K^+^-ATPase for the corresponding dose groups between HRPCM and ARPCM.

#### Differences of protein and mRNA for SGLT1 and GLUT2 in J-Pd and ileum

Research shows that the activity of SGLT1 and GLUT2 severe affect the absorption and metabolism of glucose in the small intestine (Grefner et al. 2015; Chen, Shergis, et al. 2016; Chen, Tuo, et al. 2016). In this study, the different administration groups contributed to upregulated the mRNA and protein expression of SGLT1 and GLUT2. Differences to varying degrees were found between SQD model and the remaining administration groups (*p* < 0.05 or *p* < 0.01; Figure 9), except for SGLT1 protein and GLUT2 proteins expressions for ARPCM (6.3 g/kg) in J-Pd (*p* > 0.05; Figure 9(C,E)), SGLT1 protein and mRNA expression for ARPCM (6.3 g/kg) in the ileum (*p* > 0.05; Figure 9(H,J)), and GLUT2 protein expression for ARPCM (12.6 and 6.3 g/kg) in the ileum (*p* > 0.05; Figure 9(I)). Compared with corresponding dose groups between HRPCM and ARPCM, the protein and mRNA expression of SGLT1 and GLUT2 in respective dose groups of HRPCM was all higher than that in the corresponding dose groups of ARPCM, except for GLUT2 protein expression of ARPCM (12.6 and 6.3 g/kg) in the ileum (Figure 9(I)). However, in terms of the respective dose groups of ARPCM, these upward changes in the corresponding dose groups of HRPCM showed different levels of statistical significance. Specifically, a significant difference existed in the protein and mRNA expression of SGLT1 in J-Pd between HRPCM (12.6 and 6.3 g/kg) and ARPCM (12.6 and 6.3 g/kg), respectively (*p* < 0.05 or *p* < 0.01; Figure 9(B,C)) but not between HRPCM (18.9 g/kg) and ARPCM (18.9 g/kg) (*p* > 0.05; Figure 9(B,C)). A significant difference existed in the protein expression of GLUT2 in J-Pd between HRPCM (18.9 g/kg) and ARPCM (18.9 g/kg) (*p* < 0.05; Figure 9(D)) but not between HRPCM (12.6 and 6.3 g/kg) and ARPCM (12.6 and 6.3 g/kg) (*p* > 0.05; Figure 9(B,C)). A significant difference existed in GLUT2 mRNA expression in J-Pd for the corresponding dose groups between HRPCM (18.9 g/kg) and ARPCM (18.9 g/kg) (*p* < 0.05; Figure 9(D)). A significant difference existed not only mRNA expressions of SGLT1 and GLUT2 but also protein expressions in the ileum (*p* < 0.05 or *p* < 0.01; Figure 9(G–J)). These results suggest that HRPCM and ARPCM were able to up-regulate protein and mRNA expression of SGLT1 and GLUT2, but the upregulation effect of HRPCM was more significant than that in ARPCM.

#### Pathological observation of stomach and each small intestinal segment

**Figure 10.**
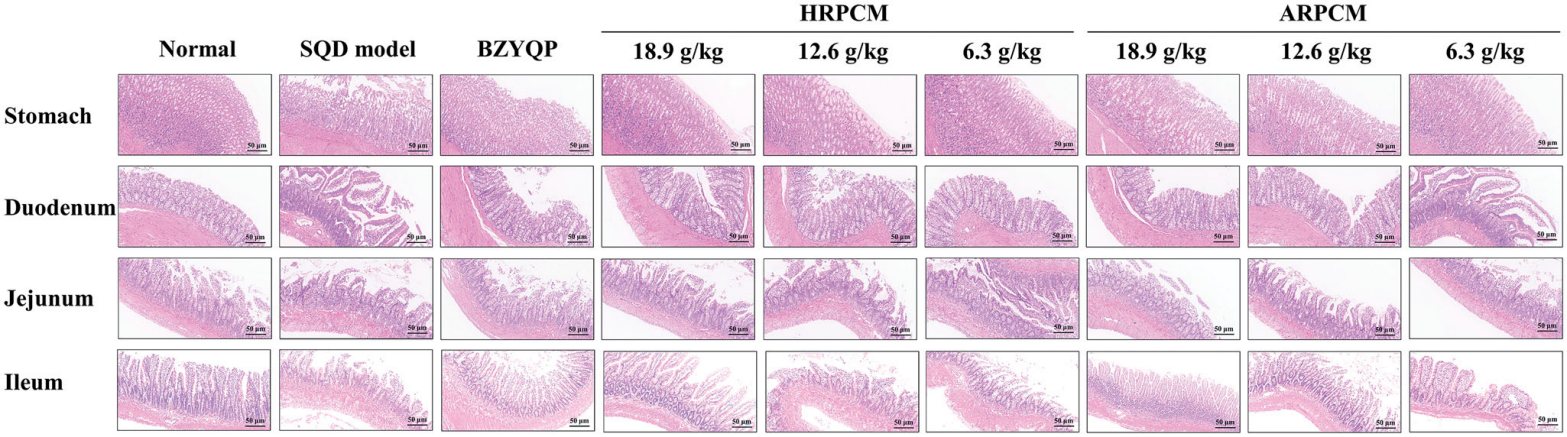
Pathological observations of the stomach, duodenum, jejunum and ileum.

The HE staining results of gastric tissues showed no significant pathological change in normal rats. In SQD model rats, the arrangement of glands was disordered, the epithelial cells were missing and the cell arrangement was disordered. However, no obvious structural-layer damage and inflammatory exudate in the mucosa were observed. Meanwhile, mucus secretion was found. The gastric tissue lesions of rats in each administration group recovered to different degrees, and neovascular tissue and cystic dilatation of the marginal regenerative gland part were visible. This finding indicated that the gastric mucosa was in the repair stage. In particular, HRPCM (18.9 g/kg) was the most significant (Figure 10).

The upper part of the small intestine starts from the pylorus of the stomach, and its lower part is connected with the large intestine via the ileocecal valve, which is divided into duodenum, jejunum and ileum. The HE staining results of duodenum, jejunum and ileum showed obvious injuries in SQD model compared with normal. The main reason may be related to the diarrhoea symptoms of SQD rats. Three parts of the small intestine had more crypt cells, the villi were shortened and indistinct, the villi tips were partially necrotic and detached, the villus epithelial cells were damaged and detached, and edoema was obvious. The intestinal glands were obviously degenerated, and the submucosa was slightly congested and severely oedematous, with a small amount of inflammatory cell infiltration. After treatment, the number, arrangement and morphological structure of glandular cells in each part of the small intestine were significantly improved, the length of villi increased, and edoema was relieved. In particular, HRPCM (18.9 g/kg) and ARPCM (18.9 g/kg) were more significant (Figure 10).

#### Comparison of efficacy between HRPCM and ARPCM on gastrointestinal function with SQD rats based on fuzzy matter-element analysis

The composite fuzzy matter-element model was built by the gastrointestinal-function-related indicators **(**Figures 8 and 9) including the expression level of d-xylose, AMS, GAS, VIP, MTL, pepsin in serum; Pepsin and H^+^/K^+^-ATPase in gastric tissue; Na^+^/K^+^-ATPase in J-Dd; as well the protein and mRNA relative expressions of SGLT1 and GLUT2 in the J-Pd and ileum. According to the methods in the fuzzy matter-element analysis (Online Supplementary Analysis 1), the closeness compound fuzzy matter-element $R_{\text{ρH}}\;$was calculated:

$$R_{\text{ρH}}=[\begin{array}{llllllllll} & M_{1} & M_{2} & M_{3} & M_{4} & M_{5} & M_{6} & M_{7} & M_{8} & M_{9}\\ {\text{ρH}}_{j} & 0.88 & 0.13 & 0.68 & 0.50 & 0.37 & 0.26 & 0.43 & 0.27 & 0.16 \end{array}]$$

The calculated closeness decreased in the order *M*_1_ > *M*_3_ > *M*_4_ > *M*_7_ > *M*_5_ > *M*_8_ > *M*_6_ > *M*_9_ > *M*_1_. Where *M*_4_, *M*_5_ and *M*_6_ are HRPCM (18.9 g/kg), HRPCM (12.6 g/kg) and HRPCM (12.6 g/kg), respectively; and *M*_4_, *M*_5_ and *M*_6_ are ARPCM (18.9 g/kg), ARPCM (12.6 g/kg) and ARPCM (12.6 g/kg), respectively. The corresponding closeness of each dose group decreased in the order *M*_4_ > *M*_7_, *M*_5_ > *M*_8_, *M*_6_ > *M*_9_, respectively. In terms of difference, the difference for each corresponding dose group between ARPCM and HRPCM were smaller, namely, $|M_{4}-M_{7}|=0.24,\;|M_{5}-M_{8}|=0.10,\;|M_{6}-M_{9}|=0.10.$ Therefore, the efficacy of HRPCM in the high-, medium- and low- dose groups were superior to that of ARPCM with regard to the regulation of gastrointestinal function in SQD.

### Differences of the pharmacological effects on regulating immune functions

#### Results of immune-organ index statistics

**Figure 11.**
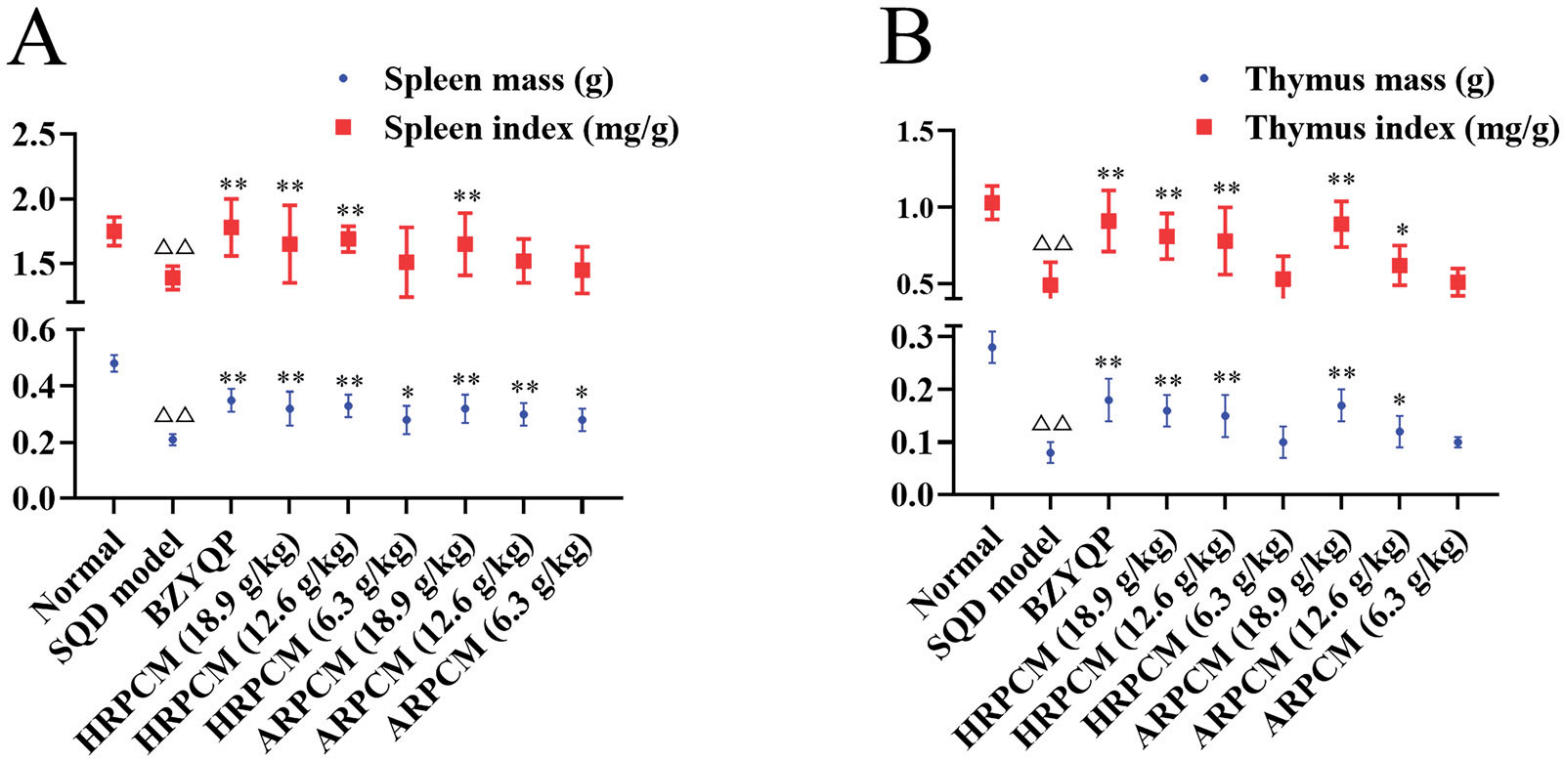
Results of spleen index and thymus index. (A) Spleen index (B) Thymus index. All data were presented as the mean ± SE (*n* = 8 ) and were analysed by ANOVA followed by Dunnett’s multiple comparison tests. ^△^*p* < 0.05 and ^△△^*p* < 0.01 when compared with normal rats. * *p* < 0.05 and **p < 0.01 when compared with SQD model rats. ^#^*p* < 0.05 and ^##^*p* < 0.05 when compared with ARPCM of corresponding doses. In other words, HRPCM (18.9 g/kg) compared with ARPCM (18.9 g/kg); HRPCM (12.6 g/kg) compared with ARPCM (12.6 g/kg); HRPCM (6.3 g/kg) compared with ARPCM (6.3 g/kg). The spleen index and thymus index for corresponding dose groups between HRPCM and ARPCM did not show statistically significant differences (*p*> 0.05).

The spleen and thymus are two main immune organs of the human body (Garcia-Calderon et al. 2018). Figure 11 shows spleen and thymus index statistics. The SQD model rats exhibited significantly lower weight and indexes for spleen and thymus than the normal rats (*p* < 0.01). After treatment, compared with the SQD model, spleen index in different administration groups expect HRPCM (6.3 g/kg) and ARPCM (12.6 and 6.3 g/kg) showed significant increases (*p* < 0.01). Thymus index in different administration groups expect HRPCM (6.3 g/kg) and ARPCM (6.3 g/kg) also showed significant increases (*p* < 0.01). However, the spleen index and thymus index for the corresponding dose groups between HRPCM and ARPCM did not show statistically significant difference. These findings indicated that HRPCM and ARPCM were capable of recovering the decrease in spleen and thymus mass owing to lesions.

#### Routine blood test results

**Figure 12.**
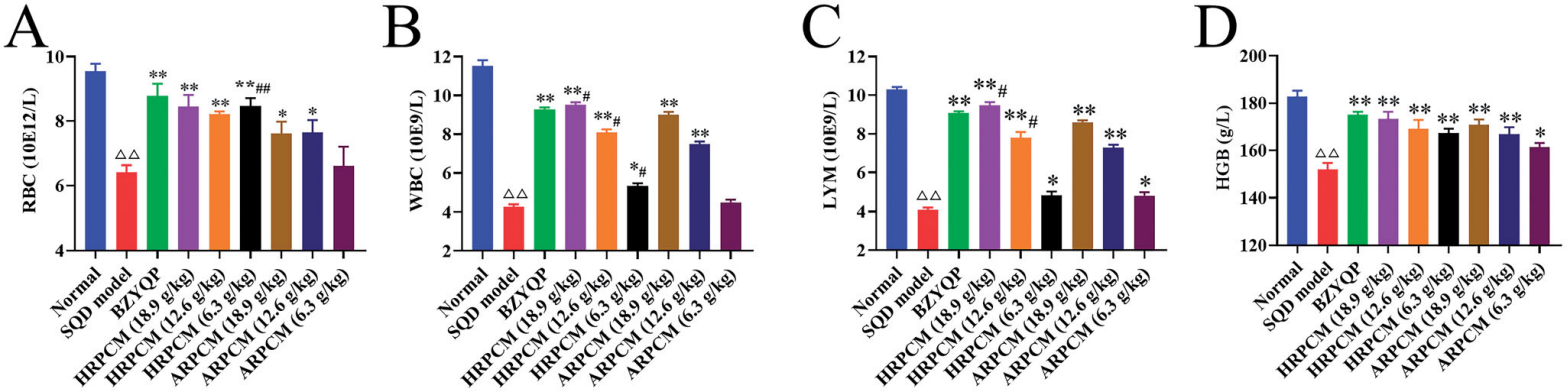
Blood routine partial indices determination results. (A) RBC (B) WBC (C) LYM (D) HGB. All data were presented as the mean ± SE (*n*= 8.) and were analysed by ANOVA followed by Dunnett’s multiple comparison tests. ^△^*p* < 0.05 and ^△△^*p* < 0.01 when compared with normal rats. *p < 0.05 and **p < 0.01 when compared with SQD model rats. ^#^*p* < 0.05 and ^##^*p* < 0.05 when compared with ARPCM of corresponding doses. In other words, HRPCM (18.9 g/kg) compared with ARPCM (18.9 g/kg); HRPCM (12.6 g/kg) compared with ARPCM (12.6 g/kg); HRPCM (6.3 g/kg) compared with ARPCM (6.3 g/kg). RBC, red blood cell; WBC, white blood cell; LYM, lymphocyte; HGB, haemoglobin.

Monitoring routine blood indicators enabled the timely control of various diseases and indirectly reflects the immune capacity of the body (Smith et al. 2009). Levels of blood routine such as RBC, WBC, LYM, and HGB significantly decreased in the SQD model rats compared with normal rats (Figure 12). These showed that the SQD model was established successfully. Levels of RBC, WBC, LYM and HGB in each administration group significantly increased (*p* < 0.05 or *p* < 0.01; Figure 12) except for WBC in ARPCM (6.3 g/kg) that had no statistical significance (*p* > 0.05; Figure 12(B)). As shown in Figure 12(A–C), RBC, WBC and LYM for the corresponding dose groups between HRPCM and ARPCM showed statistically significant differences. However, HGB for corresponding dose groups between HRPCM and ARPCM did not show statistically significant differences (Figure 12(D)).

#### Cytokines

**Figure 13.**
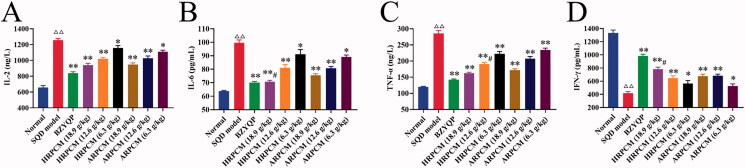
Results of immunoinflammatory indicators assay. (A) IL-2 (B) IL-6 (C) IFN-γ (D) TNF-α. All data were presented as the mean ± SE (*n*= 8) and were analysed by ANOVA followed by Dunnett’s multiple comparison tests. ^△^*p* < 0.05 and ^△△^*p* < 0.01 when compared with normal rats. *p < 0.05 and **p < 0.01 when compared with SQD model rats. ^#^*p* < 0.05 and ^##^*p* < 0.05 when compared with ARPCM of corresponding doses. In other words, HRPCM (18.9 g/kg) compared with ARPCM (18.9 g/kg); HRPCM (12.6 g/kg) compared with ARPCM (12.6 g/kg); HRPCM (6.3 g/kg) compared with ARPCM (6.3 g/kg). IL-2: interleukin-2; IL-6: interleukin-6; IFN-γ: interferon-gamma; TNF-α: tumour necrosis factor-alpha.

The serum activities of IL-2, IL-6, IFN-γ and TNF-α directly reflect the immunity of the body (Rosenberg and Calabresi 1963; De Amici et al. 2001). The results of immunoinflammatory indicators showed that compared with the normal rats, IL-2, IL-6 and TNF-α had extremely high expression but IFN-γ had extremely low expression in SQD model rats (Figure 13). These marked changes in SQD model rats showed that pathological changes occurred in the SQD rats (Wang et al. 2020). After intervention, compared with the corresponding dose groups between HRPCM and ARPCM, the significant difference was primarily shown in the high (18.9 g/kg) and middle (12.6 g/kg) dose groups. Specifically, statistically significant differences existed between HRPCM (18.9 g/kg) and ARPCM (18.9 g/kg) for IL-2, IL-6 and IFN-γ (*p* < 0.05, Figure 13(A,B,D)) and between HRPCM (12.6 g/kg) and ARPCM (12.6 g/kg) for IFN-γ and TNF-α (*p* < 0.05, Figure 13(C, D)). However, all low-dose groups had no statistically significant differences for the two drugs.

#### Humoral immunity indicators

**Figure 14.**
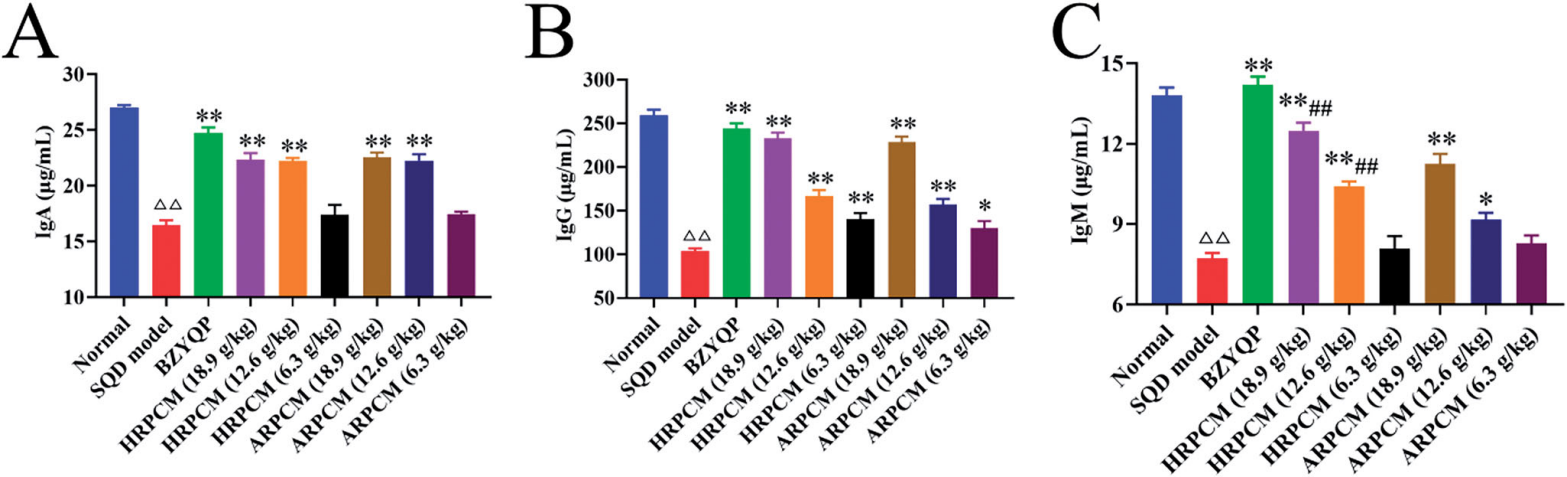
Results of humoral immunity indicators assay. (A) IgM (B) IgG (C) IgA. All data were presented as the mean ± SE (*n*= 8) and were analysed by ANOVA followed by Dunnett’s multiple comparison tests. ^△^*p* < 0.05 and ^△△^*p* < 0.01 when compared with normal rats. *p < 0.05 and **p < 0.01 when compared with SQD model rats. ^#^*p* < 0.05 and ^##^*p* < 0.05 when compared with ARPCM of corresponding doses. In other words, HRPCM (18.9 g/kg) compared with ARPCM (18.9 g/kg); HRPCM (12.6 g/kg) compared with ARPCM (12.6 g/kg); HRPCM (6.3 g/kg) compared with ARPCM (6.3 g/kg). Ig: immune globulin.

IgG, IgM and IgA are the three most common immunoglobulins in clinical practice, which are the most commonly used methods of checking humoral immune function and reflecting the humoral immune status of the body (Nikulin et al. 1981; Wright 2011). IgG, IgM and IgA levels significantly decreased in the SQD model rats (*p* < 0.01, Figure 14), indicating that the immune function of SQD rats was severely damaged. The three immunoglobulins significant increased after treatment, except for IgM and IgA in HRPCM (6.3 g/kg) and ARPCM (6.3 g/kg) (Figure 14(A,C)). Compared with the corresponding dose groups between HRPCM and ARPCM, the levels of IgM in HRPCM (18.9 and 12.6 g/kg) were significantly higher than in ARPCM (18.9 and 12.6 g/kg), respectively (*p* < 0.01; Figure 14(C)).

#### Cellular immunity

**Figure 15.**
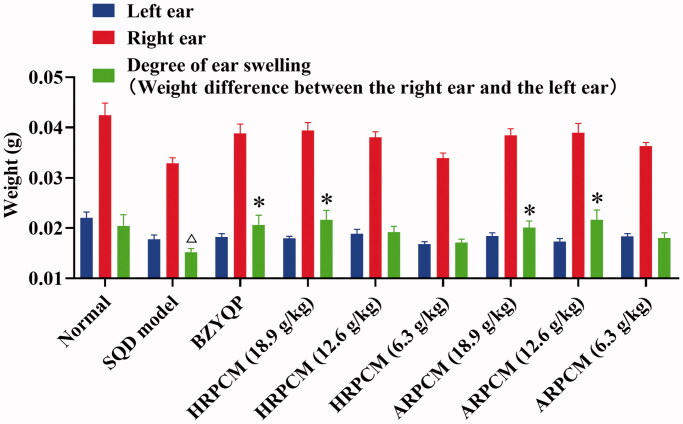
Effect of each administration group on DTH in rats. All data were presented as the mean ± SE (*n*= 8) and were analysed by ANOVA followed by Dunnett’s multiple comparison tests. ^△^*p* < 0.05 and ^△△^*p* < 0.01 when compared with normal rats. *p < 0.05 and **p < 0.01 when compared with SQD model rats. ^#^*p* < 0.05 and ^##^*p* < 0.05 when compared with ARPCM of corresponding doses. In other words, HRPCM (18.9 g/kg) compared with ARPCM (18.9 g/kg); HRPCM (12.6 g/kg) compared with ARPCM (12.6 g/kg); HRPCM (6.3 g/kg) compared with ARPCM (6.3 g/kg). The spleen index and thymus index for corresponding dose groups between HRPCM and ARPCM did not show statistically significant differences (*p* > 0.05). The DTH for corresponding dose groups between HRPCM and ARPCM did not show statistically significant differences (*p* > 0.05). DTH: delayed type hypersensitivity.

According to the literature, after applying DNFB (a hapten) solution to the abdominal wall of rats, DNFB is able to bind with skin proteins forming a complete antigen. T lymphocytes then stimulate to proliferate into sensitized lymphocytes. DTH occurs when DNFB solution is applied to the skin again 4-7 days later (Muthana et al. 2015). Generally, the degree of ear swelling is the weight difference in the left and right ears, representing DTH. In this study, DNFB solution was applied to the abdominal wall of rats. Compared with normal rats, the degree of ear swelling in the SQD model rats was significantly decreased **(***p* < 0.01; Figure 15), indicating that the SQD model rats suffered from weakened immunity. Meanwhile, the degree of ear swelling after treatment with HRPCM (18.9 g/kg) and ARPCM (18.9 and 12.6 g/kg) was significantly higher in normal than in SQD model rats in general (*p* < 0.05 or *p* < 0.01; Figure 15). These results showed that HRPCM and ARPCM enhanced DTH in rats depending on the dose administered, with the higher dose group (18.9 g/kg) exerting a more significant effect.

#### Relative expression of IL-6 mRNA and INF-γ mRNA in spleen

**Figure 16.**
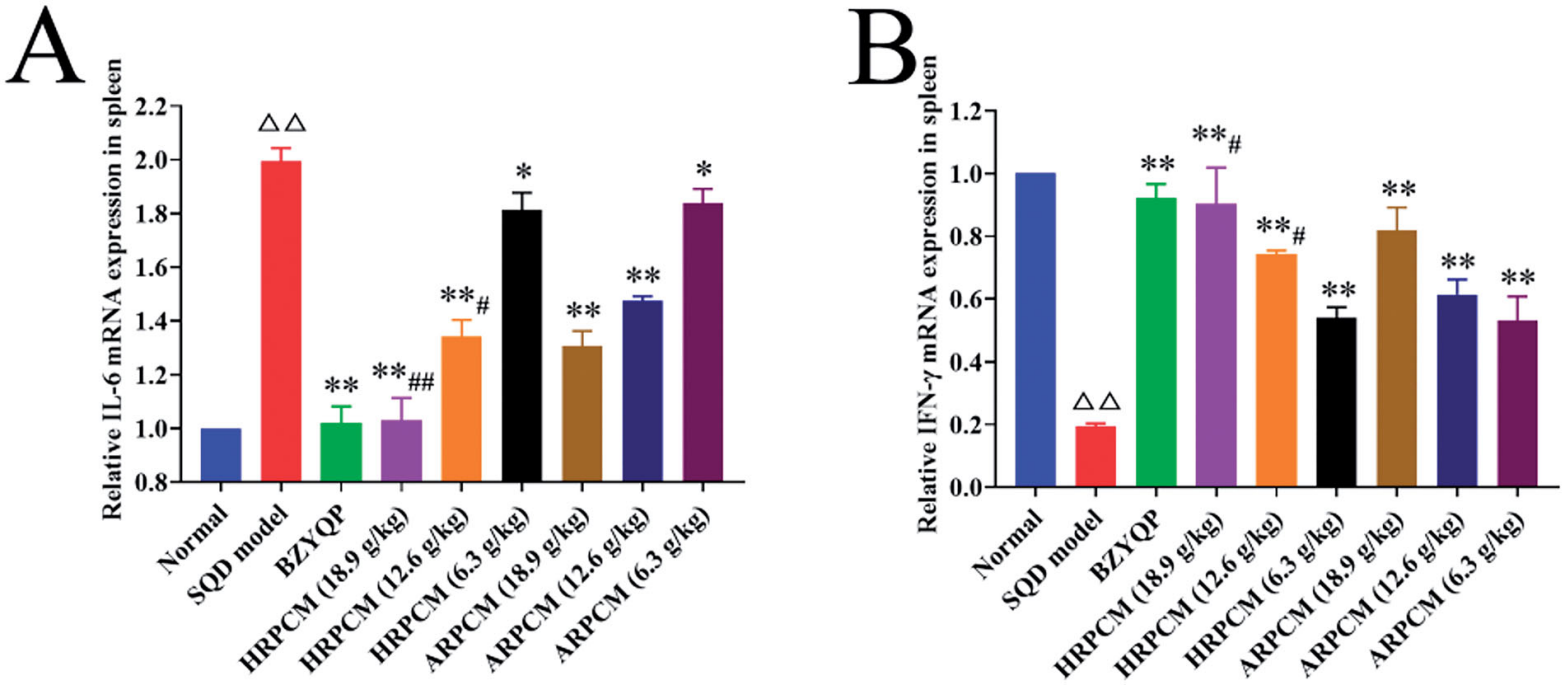
Relative IL-6 mRNA and IFN-γ mRNA expression in spleen. All data were presented as the mean ± SE (*n*= 8) and were analysed by ANOVA followed by Dunnett’s multiple comparison tests. ^△^*p* < 0.05 and ^△△^*p* < 0.01 when compared with normal rats. *p < 0.05 and **p < 0.01 when compared with SQD model rats. ^#^*p* < 0.05 and ^##^*p* < 0.05 when compared with ARPCM of corresponding doses. In other words, HRPCM (18.9 g/kg) compared with ARPCM (18.9 g/kg); HRPCM (12.6 g/kg) compared with ARPCM (12.6 g/kg); HRPCM (6.3 g/kg) compared with ARPCM (6.3 g/kg).

Both IL-6 and IFN-γ were pleiotropic mediators of inflammation and immunity. The amelioration of IFN-γ and IL-6 exerts a synergistic dampening effect on inflammation (Mortensen et al. 2014; Alvarado et al. 2019). Therefore, the expression levels of IL-6 mRNA and IFN-γ mRNA in the spleen were further determined. The relative expression of IL-6 mRNA was significantly upregulated, and the relative expression of IFN-γ mRNA was significantly downregulated in SQD model, statistical significance existed compared with the normal group (*p* < 0.01; Figure 16). After drug intervention, HRPCM and ARPCM contributed to regulating the abnormal levels of IL-6 mRNA and IFN-γ mRNA back to the normal level. These results revealed the effectiveness of HRPCM and ARPCM in SQD rats. Compared with the corresponding dose groups between HRPCM and ARPCM, the significant difference primarily was found in the high (18.9 g/kg) and middle (12.6 g/kg) dose groups, because all low-dose groups had no statistically significant difference for the two drugs (*p* > 0.05; Figure 16).

**Figure 17.**
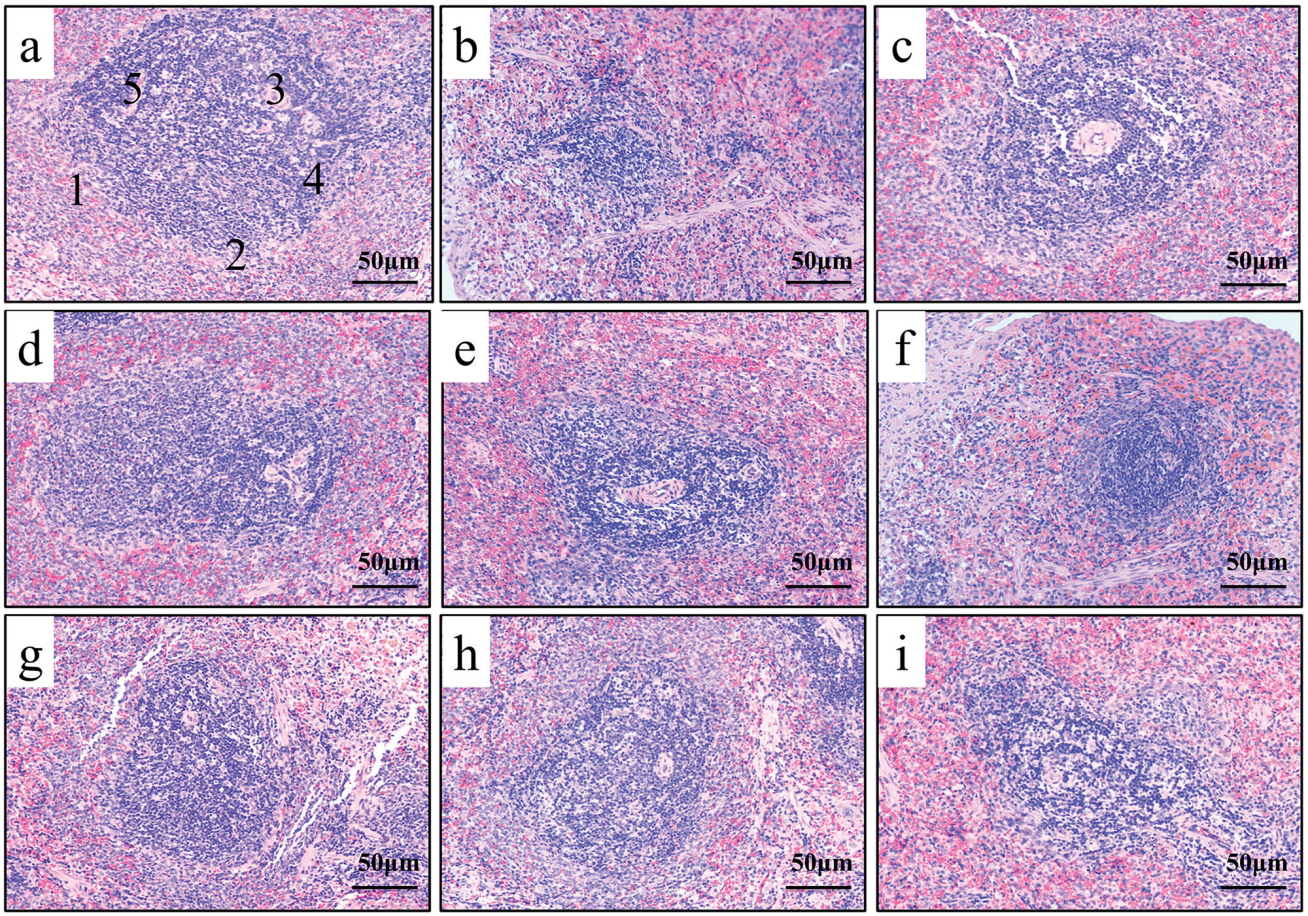
Pathological observation of spleen (200x); (a) normal; (b) SQD model; (c) BZYQP; (d) HRPCM (18.9 g/kg); (e) HRPCM (12.6 g/kg); (f) HRPCM (6.3 g/kg); (g) ARPCM (18.9 g/kg); (h) ARPCM (12.6 g/kg); (i) ARPCM (6.3 g/kg); (1) Red pulp; (2) White pulp; (3) Germinal centre; (4) Lymphocytes; (5) Splenic corpuscle.

#### Results of spleen pathological observation

The spleen, the largest secondary lymphoid organ, is an important site for initiating immune responses against systemic pathogens (Liu et al. 2020). According to Figure 17, in the spleen sections of the SQD model rats, the distribution of red pulp and white pulp was irregular, the boundary was unclear, the shape of splenic corpuscles was irregular and the arrangement was irregular, the structure of white pulp was destroyed, the number of lymphocytes was reduced and the number of germinal centres was reduced or atrophied compared with the normal rats. Splenic corpuscles in HRPCM (18.9 g/kg) and ARPCM (18.9 g/kg) were relatively uniform in shape and regular in the arrangement. The boundaries between red pulp and white pulp were visible, their respective areas were obvious, and the number of lymphocytes was greater. Compared with the corresponding low-dose groups (6.3 g/kg), the boundaries of red pulp and white pulp in the HRPCM (12.6 g/kg) and ARPCM (12.6 g/kg) were clearer, the lymphocytes in the white pulp were closer and the germinating centre was obvious. The splenic corpuscles of the HRPCM (6.3 g/kg) and ARPCM (6.3 g/kg) were blurred and abnormal in shape and structure, the boundary between white pulp and red pulp was unclear, lymphocytes was fewer and disordered, and the number of germinating centres was reduced. Overall, HRPCM and ARPCM were capable of improving the spleen pathology with SQD rats, but the intervention effect in HRPCM (6.3 g/kg) and ARPCM (6.3 g/kg) was not as good as that in HRPCM (18.9 and 12.6 g/kg), and ARPCM (18.9 and 12.6 g/kg), respectively.

#### Comparison of efficacy between HRPCM and ARPCM on regulating immune functions with SQD rats based on fuzzy matter-element analysis

The composite fuzzy matter-element model was built by the immune-regulation-related indicators **(**Figures 11–16), including the expression level of RBC, WBC, LYM, HGB in blood; the expression level of IL-2, IL-6, IFN-γ, TNF-α, IgM, IgA, IgG in serum; the DTH; as well the mRNA relative expressions of IL-6 and IFN-γ in spleen. According to the methods in the fuzzy matter-element analysis (Online Supplementary Analysis 2), the closeness compound fuzzy matter-element $R_{\text{ρH}}\;$was calculated:

$$R_{\text{ρH}}=[\begin{array}{cccccccccc} & M_{1} & M_{2} & M_{3} & M_{4} & M_{5} & M_{6} & M_{7} & M_{8} & M_{9}\\ {\text{ρH}}_{j} & 0.94 & 0.26 & 0.64 & 0.60 & 0.46 & 0.35 & 0.55 & 0.45 & 0.33 \end{array}]$$

The calculated closeness decreased in the order *M*_1_>*M*_3_>*M*_4_>*M*_7_>*M*_5_>*M*_8_>*M*_6_>*M*_9_>*M*_2_. In terms of difference, each corresponding dose groups between ARPCM and HRPCM were smaller, namely: $|M_{4}-M_{7}|=0.05,\;|M_{5}-M_{8}|=0.01,\;|M_{6}-M_{9}|=0.02.$ Therefore, it can be determined that in high dose group of the two drugs, namely 1.89 g/kg, the efficacy on regulating the immune function of HRPCM is stronger than ARPCM. In terms of the medium- and low-dose groups, the effect of HRPCM was slightly stronger than ARPCM.

## Discussion

As two traditional Chinese herbs, HRPCM and ARPCM have long been used to treat SQD. TCM theory holds that spleen-qi in the body contributes to improving the function of digestion and absorption, absorbing nutrients and indirectly improving immunity (Wang X et al. 2020). Famous compound prescriptions such as Bu-Zhong-Yi-Qi-Pill (BZYQP) and Si-Jun-Zi powder (Zheng et al. 2014; Kan and Qu 2021) are mostly used to treat the diseases such as digestive function disorders (Yu et al. 2017), improving nutritional status (Yu et al. 2017), decreased immune function (Wang et al. 2017), diarrhoea, rectal prolapse and uterine prolapse (Chen, Shergis, et al. 2016; Chen, Tuo, et al. 2016) caused by SQD syndrome, whereas single-herb medicine such as HR, AR, Rhizoma Atractylodis Macrocephalae and Radix Ginseng are primarily used in the same diseases, too (Niu et al. 2019; Yang et al. 2019). BZYQP is described in the book ‘Treatise on Spleen and Stomach (Pi-Wei-Lun)’ written by Dong-Yuan Li, a member of the ‘four great doctors of the Jin and Yuan period’ in China (1271–1368, B.C.) (Xu et al. 2020). In this study, BZYQP was selected as the positive-control drug based on its efficacy and pharmacological effects. SQD syndrome is one of the common TCM diseases in the clinical setting. The establishment of SQD animal models is highly significant for the study of spleen- and stomach- related diseases. In this study, the comprehensive method of dietary disorder and bitter-cold diarrhoea (giving purgative Chinese herbal medicine) combined with excessive fatigue method used in the previous experiments was adopted to establish a SQD rat model (Yang et al. 2019). This comprehensive method is considered as one of the best animal models of SQD (Zhong et al. 2022). We found the general signs of SQD rats were in line with the ‘Chinese Medicine Deficiency Syndrome Reference Standard’ and the SQD model standard established by the Spleen and Stomach Disease Branch of the Chinese Society of TCM (Gao et al. 2009; Huang et al. 2020). TCM theory also holds that the spleen in the will (meaning Zhi in TCM) is thinking, which is a state of human mental thinking and consciousness activities (Ouyang et al. 1998). When the spleen-qi is deficiency, it affects the metaplasia of qi and blood, the thinking consciousness activities are in a pathological state, and the capacities of learning and memory are bound to slow down (Ji et al. 2003). The Morris water maze is primarily applied in behavioural research to objectively evaluate spatial learning and cognitive capacities (Tucker et al. 2018). Blood routine test contributes to controlling the occurrence and changes of diseases in the body in time (Hawkes et al. 2018). D-Xylose, a kind of pentose, is absorbed in the small intestine after oral administration, which reflects the absorption function of the small intestine (Rubino et al. 2006). In the current work, we found that the differences were statistically significant between the SQD modelling rats and the normal rats in terms of Morris tests, routine blood indices, body weight, rectal temperature and serum D-Xylose content, which showed that the SQD model was successfully established. Fifteen days after administration, the body weight and rectal temperature in rats of each administration group were significantly upregulated. The pathological symptoms of rats in the administration groups gradually normalized, in particular, the symptoms of BZYQP and HRPCM (18.9 g/kg) rats improved significantly, proving that the previous model was successful and reliable.

According to the TCM theory, SQD impairs gastrointestinal function and reduces the ability to obtain nutrients, which then leads to the decline in immune function (Shang et al. 2013). In this study, SQD model rats not only showed gastrointestinal dysfunction, but also showed decreased immune function. After treatment with HRPCM and APPCM, the gastrointestinal and immune functions exhibited varying degrees of recovery. Differences were primarily reflected in regulating of different indicators by HRPCM and ARPCM in different dose groups. Results showed that the difference indices on regulating gastrointestinal function were D-xylose, MTL, GAS in serum, mRNA and proteins expression of SGLT1 and GLUT2 in small intestine between HRPCM and ARPCM, whereas the difference indices on regulating immune function were IL-6, IFN-γ, TNF-α and IgM in serum, as well as Il-6 mRNA and IFN-γ mRNA in spleen. Moreover, the results of fuzzy matter element analysis showed that HRPCM (18.9, 12.6 and 6.3 g/kg) was higher than the corresponding dose groups of ARPCM in regulating the gastrointestinal function of SQD rats. In regulating the immune function, the high-dose group of the two drugs, i.e., 1.89 g/kg, the efficacy of HRPCM was stronger than ARPCM. In terms of the medium- and low-dose groups (12.6 and 6.3 g/kg), the effect of HRPCM was slightly stronger than ARPCM. This relevance was consistent with the quantitative-effect relationship of TCM (Zhu et al. 2019). Previous research showed that the gastrointestinal dysfunction is associated with the development of metabolic system, early malnutrition, and decreased immune function (He et al. 2009). Therefore, it is necessary to regulate the disordered gastrointestinal function for better access to nutrition and improve immunity. This theory is consistent with the principle of TCM treatment of SQD syndrome. From this, we assume that the difference indices on regulating gastrointestinal function might have some connection with the difference indices on regulating immune function. This assumption have confirmed by previous studies. The expression level of D-xylose in serum is negatively correlated with the antigen-specific inflammatory reaction (Antunes et al. 2009). Meanwhile, when D-xylose absorption is reduced, the body is prone to iron deficiency anaemia and hypoproteinaemia (Savilahti 2000). Furthermore, another study has found that the expression level of MTL and GAS were used to assess gastrointestinal autonomic nerve dysfunction. The expression levels of MTL and GAS in serum have links with the expression levels IL-6, IgG, IgA and IgM (Zhou and Wang 2021). Nutrients such as glucose and amino acids are primarily absorbed in the small intestine. Glucose enters the enterocyte via apical SGLT1 and exits enterocytes via the basolateral GLUT-2 (Rao 2004). If the expression of SGLT1 and GLUT2 is unbalanced, it will affect the absorption of nutrients in the small intestine, and then affect the immune function (Engevik and Engevik 2021). In this study, we verified that HRPCM is more effective than ARPCM on regulating gastrointestinal function and immune function with SQD. The main reason is that HRPCM had a stronger regulatory ability for the above-mentioned different indices than ARPCM.

## Conclusions

This study demonstrated the difference indicators of HRPCM and ARPCM on regulating gastrointestinal and immune functions with SQD rats based on existing disquisitive indicators. HRPCM was more effective than ARPCM at the same administered dose on regulating gastrointestinal and immune functions with SQD. Based on the shortcomings of the above study, we propose the following prospects. The correlations of the different indices should be further studied between the immune function and gastrointestinal function of HRPCM and ARPCM. The efficacy difference in HRPCM and ARPCM on the intervention of SQD rats may be discussed by studying the related metabolic. The efficacy difference in HRPCM and ARPCM on the intervention of SQD rats may be discussed by studying the related metabolic pathways. This experiment preliminarily verified the efficacy difference between HRPCM and ARPCM on intervening gastrointestinal function and immunoregulatory function of SQD. The comparison of the differences and contents of various active compositions (such as polysaccharides, flavonoids and saponins.) in HRPCM and ARPCM can be regarded as a future research focus.

## Supplementary Material

Supplemental Material

Supplemental Material

## Data Availability

The data presented in this study are available from the authors.
