# Supplementary material for: Comparative study on the gastrointestinal- and immune- regulation functions of Hedysari Radix Paeparata Cum Melle and Astragali Radix Praeparata cum Melle in rats with spleen-qi deficiency, based on fuzzy matter-element analysis
Source: Pharm Biol. 2022 Jun 28;60(1):1237–54. doi: 10.1080/13880209.2022.2086990 (PMC9246251; doi:10.1080/13880209.2022.2086990)
Supplement: Supplemental Material [file IPHB_A_2086990_SM7799.docx]

**Supplementary method 1**

**The methods of HPLC analysis.**

- 1. **HRPCM**

The compositions of HRPCM were characterized using Agilent 1260 HPLC carried out on the chromatographic column of Agilent HC-C 18 (4.6 mm × 250 mm, 5 μm) at 30 ℃. The mobile phase comparised acetonitrile (A) and 0.01% phosphorus acid aqueous solution (B) through the following gradient program: 0–5 min, 2–5% A; 5–20 min, 5–16% A; 20–30 min, 16–23% A; 30–35 min, 23–25% A; 35–40 min, 25–33% A; 40–45 min, 33–36% A; 45–60%, 36–50% A; 60–70 min, 50–75% A; 70–75 min, 75% A. The injection volume was set to 5 μL, the flow rate was maintained at 1 mL/min, and the detection wavelength was set at 254 nm. The reference substances were vanillic acid, calycosin-7-O-β-D-glucoside, ononin, calycosin, and formononetin (all purity > 98%; batch numbers PS000459, PS000687, PS000671, PS010251, and PS000674, respectively. Push Bio-technology Co., Ltd., Chengdu, China). All reference substances were dissolved under ultrasonication in methanol and prepared as single-component reference solution with mass concentrations of 0.84 mg/mL, 0.62 mg/mL, 1.14 mg/mL, 1.41 mg/mL, and 1.43 mg/mL. About 2 mL of single-component reference solution for the five reference substances were accurately absorbed, and the volume was fixed to 10 mL with methanol for storage of the mixed reference solution. HRPCM powder (3.00 g) was dissolved in 30 mL of methanol, heated in a 70 ℃ water bath for reflux extraction for 1 h, and filtered under reduced pressure. After concentrating the filtrate, a fixed volume was poured into a 10 mL measuring bottle for storage.

- 1. **ARPCM**

The flavonoids and saponins of ARPCM were detected using Agilent 1260 HPLC-DAD and HPLC-ELSDA, respectively. The same chromatographic column (Agilent HC-C18; 4.6 mm × 250 mm, 5 μm) was used, and the same flow rate was maintained at 1 mL/min for DAD detector and ELSDA detector. Meanwhile, differences existed in the mobile phase and detection methods for the two detectors. In HPLC-DAD analysis, the mobile phase comprised acetonitrile (A) and 0.2% formic acid aqueous solution (B), and the following gradient program was used: 0–5 min, 5%–13% A; 5–10 mi, 13%–21% A; 10–23 min, 21%–37% A; 23–33 min, 37%–53% A; 33–43 min, 53%–69% A; 43–45 min, 69%–100% A.. The injection volume, detection wavelength, and column temperature were set at 5 μL, 254 nm, and 30 ℃, respectively. In HPLC-ELSDA analysis, the mobile phase comprised acetonitrile (A) and ultrapure water (B), and the following gradient program was used: 0–5 min, 5%–13% A; 5–10 min, 13%–21% A; 10–23 min, 21%–37% A; 23–33 min, 37%–53% A; 33–43 min, 53%–69% A; 43–50 min, 69%–100% A.. The nitrogen gas flow, drift tube temperature, and atomisation temperature were set at 2.5 L/min, 105 ℃, and 30 ℃, respectively. Calycosin-7-O-β-D-glucoside, ononin, calycosin, formononetin, astragalosideⅠ, astragaloside II, astragaloside Ⅲ, and astragaloside IV were selected as the reference substances (purity > 98%; batch numbers PS000687, PS000671, PS010251, PS000674, PS000459, PS000462, PS200514-03, and PS010428, respectively; Push Bio-technology Co., Ltd., Chengdu, China). All reference substances were dissolved under ultrasonication in methanol and prepared as single-component reference solution with mass concentrations of 0.403, 0.325, 0.034, 0.104, 7.278, 1.022, 1.010, and 1.002 mg/mL. About 2 mL of single-component reference solution in calycosin-7-O-β-D-glucoside, ononin, calycosin, and formononetin were accurately absorbed, and the volume was fixed to 10 mL with methanol for storage of the mixed reference solution. Astragaloside I, astragaloside II, astragaloside Ⅲ, and astragaloside IV reference solutions were not prepared as a mixed standard solution. ARPCM powder (4.00 g) was dissolved in 40 mL of methanol, heated in a 70 ℃ water bath for reflux extraction for 1 h, and filtered under reduced pressure. After concentrating the filtrate, a fixed volume was poured into 10 mL measuring bottle for storage. We performed heating reflux extraction twice for 2 h each time, filtration, and reduction and recycling in methanol. The residue was extracted with water-saturated n-butanol twice (40 mL each time), and n-butanol was combined with ammonia test solution for washing twice (40 mL each time). The ammonia test solution was discarded, and n-butanol was recovered by decompression. The residue was dissolved with the appropriate amount of methanol, and the volume was fixed in a 5 mL volumetric flask, which was shaken well and passed through a 0.45 μm filter membrane.

**Supplementary method 2**

**The methods Behavior experiments, D-xylose test and blood routine test in modelling process**

**1.1 Morris water maze test**

The water maze comprised a circular pool 150 cm in diameter and 50 cm in height. At the same time, a platform with a diameter of 6 cm and a height of 14 cm, as well as a computerised video-tracking system including cameras, video recorders, computer monitors, and analysis software, was also added to the water maze together. The circular pool was divided into four quadrants (NE, SE, SW, and NW) in the southeast and northwest and was filled with water at a temperature of 23±2 ℃. The tests contained the oriented-navigation test and the s patial probe test (Gong et al., 2020). During the oriented navigation test, the rats were placed in the water facing the pool wall. Escape latency was recorded by the computer as the time that a rat spent to reach the platform. The test was started on the 11th day of modelling for 10 rats in normal and 40 rats randomly selected from SQD modelling. The test lasted for 4 days. Then, the average value was taken and the swimming trajectory graph was observed. The platform was taken away for the spatial probe test on the 15th day of modelling (Bromley-Brits et al., 2011; Lu et al., 2012). The number of times crossing the platform area, target quadrant retention time and percentage were recorded by the computer within 120 s.

**1.2 D-Xylose absorption test and blood routine test**

The SQD modelling rats were fasted overnight on the 15th day of modelling. Six rats were randomly selected from the normal and SQD modelling respectively on the 16th morning. Then, 5% D-xylose solution with a dosage of 10 mL/kg was given to rats by ig. After 1 h, rats were anesthetized with 10% chloral hydrate solution. After collecting 1.5 mL of blood sample from the abdominal aorta into non-anticoagulated vacuum-blood-collection tubes and 1.0 mL of blood sample was collected into anticoagulated vacuum blood-collection tubes, D-xylose content in serum obtained from non-anticoagulant blood was determined by the phloroglucinol method (Xiao et al., 2021). Routine blood tests including RBC, HGB, WBC, LYM, and PLT, were analysed with a hematology analyser.
