# Supplementary material for: Comparative study on the gastrointestinal- and immune- regulation functions of Hedysari Radix Paeparata Cum Melle and Astragali Radix Praeparata cum Melle in rats with spleen-qi deficiency, based on fuzzy matter-element analysis
Source: Pharm Biol. 2022 Jun 28;60(1):1237–54. doi: 10.1080/13880209.2022.2086990 (PMC9246251; doi:10.1080/13880209.2022.2086990)
Supplement: Supplemental Material [file IPHB_A_2086990_SM7795.docx]

**Supplementary analysis 1**

**Comparison of efficacy between HRPCM and ARPCM on gastrointestinal function with SQD rats based on fuzzy matter-element analysis.**

The composite fuzzy matter-element model was built by the gastrointestinal-function-related indicators including the expression level of D-xylose, AMS, GAS, VIP, MTL, pepsin in serum; Pepsin and H^+^/K^+^-ATPase in [gastric](javascript:;) [tissue](javascript:;); Na^+^/K^+^-ATPase in J-Dd; as well the protein and mRNA relative expressions of SGLT1 and GLUT2 in the J-Pd and ileum, namely,

$$R_{9-16}=\left[ \begin{matrix} & M_{1} & M_{2} & M_{3} & M_{4} & M_{5} & M_{6} & M_{7} & M_{8} & M_{9} \\ C_{1} & 8.62 & 4.36 & 7.99 & 6.19 & 5.36 & 5.63 & 6.01 & 7.53 & 5.14 \\ C_{2} & 213.27 & 76.57 & 223.27 & 201.21 & 173.09 & 100.95 & 193.27 & 140.86 & 91.04 \\ C_{3} & 76.55 & 33.47 & 77.73 & 60.24 & 42.80 & 35.63 & 63.75 & 58.51 & 43.06 \\ C_{4} & 452.73 & 191.35 & 378.07 & 343.70 & 298.56 & 262.88 & 304.29 & 264.88 & 225.21 \\ C_{5} & 18.97 & 3.75 & 16.09 & 10.49 & 11.03 & 7.11 & 9.75 & 11.49 & 6.64 \\ C_{6} & 57.79 & 140.22 & 73.12 & 87.46 & 106.21 & 124.04 & 93.78 & 103.97 & 122.66 \\ C_{7} & 1.49 & 3.70 & 1.15 & 1.80 & 2.56 & 3.28 & 1.52 & 2.46 & 3.02 \\ C_{8} & 306.11 & 85.85 & 300.97 & 199.76 & 137.36 & 113.06 & 232.72 & 129.40 & 107.32 \\ C_{9} & 1.63 & 0.29 & 1.27 & 0.87 & 0.62 & 0.47 & 0.82 & 0.49 & 0.31 \\ C_{10} & 1.63 & 0.28 & 1.18 & 0.89 & 0.52 & 0.44 & 0.70 & 0.53 & 0.31 \\ C_{11} & 1.00 & 0.07 & 0.93 & 0.90 & 0.81 & 0.60 & 0.82 & 0.55 & 0.21 \\ C_{12} & 1.00 & 0.09 & 0.91 & 0.88 & 0.66 & 0.49 & 0.77 & 0.43 & 0.19 \\ C_{13} & 1.09 & 0.19 & 1.01 & 1.04 & 0.79 & 0.59 & 0.70 & 0.50 & 0.27 \\ C_{14} & 1.08 & 0.28 & 1.07 & 1.05 & 1.02 & 0.76 & 0.92 & 0.28 & 0.20 \\ C_{15} & 1.00 & 0.17 & 0.92 & 0.82 & 0.65 & 0.34 & 0.59 & 0.48 & 0.23 \\ C_{16} & 1.00 & 0.26 & 0.90 & 0.83 & 0.67 & 0.48 & 0.64 & 0.41 & 0.28 \end{matrix} \right]$$

Amongst the gastrointestinal-function-related indicators, C_7_ (VIP) and C_8_ (H^+^/K^+^-ATPase) were smaller and more optimal but the others were larger and more optimal. Therefore, the superior membership fuzzy matter-element ${R'}_{9-16}$was built according to the formula (2) and the matrix $R_{9-16}$.

$${R'}_{9-16}=\left[ \begin{matrix} & M_{1} & M_{2} & M_{3} & M_{4} & M_{5} & M_{6} & M_{7} & M_{8} & M_{9} \\ C_{1} & 1.00 & 0.51 & 0.93 & 0.72 & 0.62 & 0.65 & 0.70 & 0.87 & 0.60 \\ C_{2} & 0.96 & 0.34 & 1.00 & 0.90 & 0.78 & 0.45 & 0.87 & 0.63 & 0.41 \\ C_{3} & 0.98 & 0.43 & 1.00 & 0.78 & 0.55 & 0.46 & 0.82 & 0.75 & 0.55 \\ C_{4} & 1.00 & 0.42 & 0.84 & 0.76 & 0.66 & 0.58 & 0.67 & 0.59 & 0.50 \\ C_{5} & 1.00 & 0.20 & 0.85 & 0.55 & 0.58 & 0.37 & 0.51 & 0.61 & 0.35 \\ C_{6} & 1.00 & 0.41 & 0.79 & 0.66 & 0.54 & 0.47 & 0.62 & 0.56 & 0.47 \\ C_{7} & 0.77 & 0.31 & 1.00 & 0.64 & 0.45 & 0.35 & 0.76 & 0.47 & 0.38 \\ C_{8} & 1.00 & 0.28 & 0.98 & 0.65 & 0.45 & 0.37 & 0.76 & 0.42 & 0.35 \\ C_{9} & 1.00 & 0.18 & 0.78 & 0.53 & 0.38 & 0.29 & 0.51 & 0.30 & 0.19 \\ C_{10} & 1.00 & 0.17 & 0.72 & 0.54 & 0.32 & 0.27 & 0.43 & 0.32 & 0.19 \\ C_{11} & 1.00 & 0.07 & 0.93 & 0.90 & 0.81 & 0.60 & 0.82 & 0.55 & 0.21 \\ C_{12} & 1.00 & 0.09 & 0.91 & 0.88 & 0.66 & 0.49 & 0.77 & 0.43 & 0.19 \\ C_{13} & 1.00 & 0.17 & 0.92 & 0.95 & 0.72 & 0.54 & 0.64 & 0.46 & 0.25 \\ C_{14} & 1.00 & 0.25 & 0.99 & 0.97 & 0.94 & 0.70 & 0.85 & 0.26 & 0.18 \\ C_{15} & 1.00 & 0.17 & 0.92 & 0.82 & 0.65 & 0.34 & 0.59 & 0.48 & 0.23 \\ C_{16} & 1.00 & 0.26 & 0.90 & 0.83 & 0.67 & 0.48 & 0.64 & 0.41 & 0.28 \end{matrix} \right]$$

$${R'}_{0-16}=\left[ \begin{matrix} & M_{0} \\ C_{1} & 1.00 \\ C_{2} & 1.00 \\ C_{3} & 1.00 \\ C_{4} & 1.00 \\ C_{5} & 1.00 \\ C_{6} & 1.00 \\ C_{7} & 1.00 \\ C_{8} & 1.00 \\ C_{9} & 1.00 \\ C_{10} & 1.00 \\ C_{11} & 1.00 \\ C_{12} & 1.00 \\ C_{13} & 1.00 \\ C_{14} & 1.00 \\ C_{15} & 1.00 \\ C_{16} & 1.00 \end{matrix} \right]$$

And then, the simple difference absolute-value composite fuzzy matter-element *R*_∆_ comprised the matrix *R*’0-16 and *R*’9-16 as follows.

$$R_{\Delta}=\left[ \begin{matrix} & M_{1} & M_{2} & M_{3} & M_{4} & M_{5} & M_{6} & M_{7} & M_{8} & M_{9} \\ C_{1} & 0.00 & 0.49 & 0.07 & 0.28 & 0.38 & 0.35 & 0.30 & 0.13 & 0.40 \\ C_{2} & 0.04 & 0.66 & 0.00 & 0.10 & 0.22 & 0.55 & 0.13 & 0.37 & 0.59 \\ C_{3} & 0.02 & 0.57 & 0.00 & 0.22 & 0.45 & 0.54 & 0.18 & 0.25 & 0.45 \\ C_{4} & 0.00 & 0.58 & 0.16 & 0.24 & 0.34 & 0.42 & 0.33 & 0.41 & 0.50 \\ C_{5} & 0.00 & 0.80 & 0.15 & 0.45 & 0.42 & 0.63 & 0.49 & 0.39 & 0.65 \\ C_{6} & 0.00 & 0.59 & 0.21 & 0.34 & 0.46 & 0.53 & 0.38 & 0.44 & 0.53 \\ C_{7} & 0.23 & 0.69 & 0.00 & 0.36 & 0.55 & 0.65 & 0.24 & 0.53 & 0.62 \\ C_{8} & 0.00 & 0.72 & 0.02 & 0.35 & 0.55 & 0.63 & 0.24 & 0.58 & 0.65 \\ C_{9} & 0.00 & 0.82 & 0.22 & 0.47 & 0.62 & 0.71 & 0.49 & 0.70 & 0.81 \\ C_{10} & 0.00 & 0.83 & 0.28 & 0.46 & 0.68 & 0.73 & 0.57 & 0.68 & 0.81 \\ C_{11} & 0.00 & 0.93 & 0.07 & 0.10 & 0.19 & 0.40 & 0.18 & 0.45 & 0.79 \\ C_{12} & 0.00 & 0.91 & 0.09 & 0.12 & 0.34 & 0.51 & 0.23 & 0.57 & 0.81 \\ C_{13} & 0.00 & 0.83 & 0.08 & 0.05 & 0.28 & 0.46 & 0.36 & 0.54 & 0.75 \\ C_{14} & 0.00 & 0.75 & 0.01 & 0.03 & 0.06 & 0.30 & 0.15 & 0.74 & 0.82 \\ C_{15} & 0.00 & 0.83 & 0.08 & 0.18 & 0.35 & 0.66 & 0.41 & 0.52 & 0.77 \\ C_{16} & 0.00 & 0.74 & 0.10 & 0.17 & 0.33 & 0.52 & 0.36 & 0.59 & 0.72 \end{matrix} \right]$$

Subsequently, the mean value of each evaluation indicator was calculated according to the formula (8), $X_{i}=$ 6.32, 157.06, 54.64, 302.41, 10.59, 101.03, 2.33, 179.17, 0.75, 0.72, 0.65, 0.60, 0.69, 0.74, 0.58, 0.61. The mean squared error of each evaluation indicator was calculated according to the formula (9), $D_{i}=$ 1.34, 53.03, 15.73, 75.50, 4.43, 24.62, 0.84, 79.38, 0.43, 0.42, 0.31, 0.30, 0.31, 0.36, 0.28, 0.25. The coefficient of variation of each evaluation indicator was calculated according to the formula (10), $\delta_{i}=$ 0.21, 0.34, 0.29, 0.25, 0.42, 0.24, 0.36, 0.44, 0.57, 0.58, 0.47, 0.50, 0.45, 0.49, 0.49, 0.42. The weight of each evaluation indicator was calculated according to the formula (11), $W_{i}=$ 0.03, 0.05, 0.04, 0.04, 0.06, 0.04, 0.06, 0.07, 0.09, 0.09, 0.07, 0.08, 0.07, 0.07, 0.08, 0.06. Finally, the corresponding values in Wi and $R_{\Delta}$ were substituted into the formula (13) and the closeness compound fuzzy matter-element $R_{H}$was calculated:

$$R_{H}=\left[ \begin{matrix} & M_{1} & M_{2} & M_{3} & M_{4} & M_{5} & M_{6} & M_{7} & M_{8} & M_{9} \\ & & & & & & & & & \\ H_{j} & 0.88 & 0.13 & 0.68 & 0.50 & 0.37 & 0.26 & 0.43 & 0.27 & 0.16 \end{matrix} \right]$$

The calculated closeness decreased in the order *M*_1_ > *M*_3_ > *M*_4_ > *M*_7_ > *M*_5_ > *M*_8_ > *M*_6_ > *M*_9_ > *M*_1_. Where *M*_4_, *M*_5_, and *M*_6_ are HRPCM (18.9 g/kg), HRPCM (12.6 g/kg) and HRPCM (12.6 g/kg) respectively; and *M*_4_, *M*_5_ and *M*_6_ are ARPCM (18.9 g/kg), ARPCM (12.6 g/kg) and ARPCM (12.6 g/kg) respectively. The corresponding closeness of each dose group decreased in the order *M*_4_ > *M*_7_, *M*_5_ > *M*_8_, *M*_6_ > *M*_9_, respectively. In terms of difference, the difference for each corresponding dose group between ARPCM and HRPCM were smaller, namely, $\left| M_{4}-M_{7} \right|=0.24, \left| M_{5}-M_{8} \right|=0.10, \left| M_{6}-M_{9} \right|=0.10$. Therefore, the efficacy of HRPCM in the high-, medium- and low- dose groups were superior to that of ARPCM with regard to the regulation of gastrointestinal function in SQD.

**Supplementary analysis 2**

**Comparison of efficacy between HRPCM and ARPCM on** **regulating immune functions with SQD rats based on fuzzy matter-element analysis**

The composite fuzzy matter-element model was built by the immune-regulation-related indicators **(Fig. 5**), including the expression level of RBC, WBC, LYM, HGB in blood; the expression level of IL-2, IL-6, IFN-γ, TNF-α, IgM, IgA, IgG in [serum](javascript:;); the DTH; as well the mRNA relative expressions of IL-6 and IFN-γ in spleen, namely,

$$R_{9-14}=\left[ \begin{matrix} & M_{1} & M_{2} & M_{3} & M_{4} & M_{5} & M_{6} & M_{7} & M_{8} & M_{9} \\ C_{1} & 9.54 & 6.41 & 8.78 & 8.45 & 8.22 & 8.47 & 7.62 & 7.65 & 6.61 \\ C_{2} & 11.52 & 4.27 & 9.28 & 9.50 & 8.09 & 5.33 & 8.99 & 7.48 & 4.49 \\ C_{3} & 9.84 & 4.09 & 9.08 & 9.48 & 7.80 & 4.82 & 8.60 & 7.27 & 4.81 \\ C_{4} & 182.70 & 151.90 & 175.00 & 173.33 & 169.00 & 167.29 & 170.80 & 166.90 & 161.40 \\ C_{5} & 658.23 & 1256.67 & 837.40 & 938.44 & 1020.73 & 1156.15 & 948.85 & 1025.94 & 1109.27 \\ C_{6} & 63.71 & 99.51 & 70.00 & 70.66 & 80.99 & 91.09 & 75.62 & 80.72 & 89.10 \\ C_{7} & 1330.00 & 416.88 & 982.50 & 782.50 & 644.38 & 562.50 & 676.88 & 682.50 & 528.13 \\ C_{8} & 119.91 & 285.47 & 141.90 & 162.50 & 190.74 & 222.74 & 171.70 & 207.64 & 234.03 \\ C_{9} & 13.81 & 7.72 & 14.19 & 12.47 & 10.42 & 8.08 & 11.25 & 9.17 & 8.27 \\ C_{10} & 27.03 & 16.47 & 24.74 & 22.54 & 22.26 & 17.42 & 22.34 & 22.22 & 17.38 \\ C_{11} & 258.77 & 103.84 & 243.98 & 232.80 & 166.51 & 140.37 & 228.40 & 157.10 & 129.81 \\ C_{12} & 0.020422 & 0.015152 & 0.020614 & 0.021638 & 0.019198 & 0.017087 & 0.020043 & 0.021648 & 0.018016 \\ C_{13} & 1.00 & 1.99 & 1.12 & 1.17 & 1.43 & 1.81 & 1.19 & 1.47 & 1.84 \\ C_{14} & 1.00 & 0.19 & 0.76 & 0.90 & 0.74 & 0.69 & 0.87 & 0.80 & 0.67 \end{matrix} \right]$$

Amongst the immune-regulation-related indicators, C_5_ (IL-2), C_6_ (IL-6), C_8_ (TNF-α), and C_13_ (the mRNA relative expressions of IL-6) were smaller and more optimal, whereas the others were larger and more optimal. Therefore, the superior membership fuzzy matter-element ${R'}_{9-14}$was built according to the formula (2) and the matrix $R_{9-14}$.

$${R'}_{9-14}=\left[ \begin{matrix} & M_{1} & M_{2} & M_{3} & M_{4} & M_{5} & M_{6} & M_{7} & M_{8} & M_{9} \\ C_{1} & 1.00 & 0.67 & 0.92 & 0.89 & 0.86 & 0.89 & 0.80 & 0.80 & 0.69 \\ C_{2} & 1.00 & 0.37 & 0.81 & 0.83 & 0.70 & 0.46 & 0.78 & 0.65 & 0.39 \\ C_{3} & 1.00 & 0.42 & 0.92 & 0.96 & 0.79 & 0.49 & 0.87 & 0.74 & 0.49 \\ C_{4} & 1.00 & 0.83 & 0.96 & 0.95 & 0.93 & 0.92 & 0.93 & 0.91 & 0.88 \\ C_{5} & 1.00 & 0.52 & 0.79 & 0.70 & 0.64 & 0.57 & 0.69 & 0.64 & 0.59 \\ C_{6} & 1.00 & 0.64 & 0.91 & 0.90 & 0.79 & 0.70 & 0.84 & 0.79 & 0.72 \\ C_{7} & 1.00 & 0.31 & 0.74 & 0.59 & 0.48 & 0.42 & 0.51 & 0.51 & 0.40 \\ C_{8} & 1.00 & 0.42 & 0.85 & 0.74 & 0.63 & 0.54 & 0.70 & 0.58 & 0.51 \\ C_{9} & 0.97 & 0.54 & 1.00 & 0.88 & 0.73 & 0.57 & 0.79 & 0.65 & 0.58 \\ C_{10} & 1.00 & 0.61 & 0.92 & 0.83 & 0.82 & 0.64 & 0.83 & 0.82 & 0.64 \\ C_{11} & 1.00 & 0.40 & 0.94 & 0.90 & 0.64 & 0.54 & 0.88 & 0.61 & 0.50 \\ C_{12} & 0.943388 & 0.699942 & 0.952258 & 0.999551 & 0.886846 & 0.789336 & 0.925896 & 1.000000 & 0.832255 \\ C_{13} & 1.00 & 0.50 & 0.89 & 0.86 & 0.70 & 0.55 & 0.84 & 0.68 & 0.54 \\ C_{14} & 1.00 & 0.19 & 0.76 & 0.90 & 0.74 & 0.69 & 0.87 & 0.80 & 0.67 \end{matrix} \right]$$

$${R'}_{0-14}=\left[ \begin{matrix} & M_{0} \\ C_{1} & 1.00 \\ C_{2} & 1.00 \\ C_{3} & 1.00 \\ C_{4} & 1.00 \\ C_{5} & 1.00 \\ C_{6} & 1.00 \\ C_{7} & 1.00 \\ C_{8} & 1.00 \\ C_{9} & 1.00 \\ C_{10} & 1.00 \\ C_{11} & 1.00 \\ C_{12} & 1.00 \\ C_{13} & 1.00 \\ C_{14} & 1.00 \end{matrix} \right]$$

And then, the simple difference absolute-value composite fuzzy matter-element *R*_∆_ comprised the matrix *R*’0-16 and *R*’9-16 as follows.

$$R_{\Delta}=\left[ \begin{matrix} & M_{1} & M_{2} & M_{3} & M_{4} & M_{5} & M_{6} & M_{7} & M_{8} & M_{9} \\ C_{1} & 0.00 & 0.33 & 0.08 & 0.11 & 0.14 & 0.11 & 0.20 & 0.20 & 0.31 \\ C_{2} & 0.00 & 0.63 & 0.19 & 0.17 & 0.30 & 0.54 & 0.22 & 0.35 & 0.61 \\ C_{3} & 0.00 & 0.58 & 0.08 & 0.04 & 0.21 & 0.51 & 0.13 & 0.26 & 0.51 \\ C_{4} & 0.00 & 0.17 & 0.04 & 0.05 & 0.07 & 0.08 & 0.07 & 0.09 & 0.12 \\ C_{5} & 0.00 & 0.48 & 0.21 & 0.30 & 0.36 & 0.43 & 0.31 & 0.36 & 0.41 \\ C_{6} & 0.00 & 0.36 & 0.09 & 0.10 & 0.21 & 0.30 & 0.16 & 0.21 & 0.28 \\ C_{7} & 0.00 & 0.69 & 0.26 & 0.41 & 0.52 & 0.58 & 0.49 & 0.49 & 0.60 \\ C_{8} & 0.00 & 0.58 & 0.15 & 0.26 & 0.37 & 0.46 & 0.30 & 0.42 & 0.49 \\ C_{9} & 0.03 & 0.46 & 0.00 & 0.12 & 0.27 & 0.43 & 0.21 & 0.35 & 0.42 \\ C_{10} & 0.00 & 0.39 & 0.08 & 0.17 & 0.18 & 0.36 & 0.17 & 0.18 & 0.36 \\ C_{11} & 0.00 & 0.60 & 0.06 & 0.10 & 0.36 & 0.46 & 0.12 & 0.39 & 0.50 \\ C_{12} & 0.06 & 0.30 & 0.05 & 0.00 & 0.11 & 0.21 & 0.07 & 0.00 & 0.17 \\ C_{13} & 0.00 & 0.50 & 0.11 & 0.14 & 0.30 & 0.45 & 0.16 & 0.32 & 0.46 \\ C_{14} & 0.00 & 0.81 & 0.24 & 0.10 & 0.26 & 0.31 & 0.13 & 0.20 & 0.33 \end{matrix} \right]$$

Subsequently, the mean value of each evaluation indicator was calculated according to the formula (8),$X_{i}=$ 7.97, 7.66, 7.31, 168.70, 994.63, 80.16, 659.53, 192.96, 10.60, 21.38, 184.62, 0.02, 1.45, 0.74. The mean squared error of each evaluation indicator was calculated according to the formula (9), $D_{i}=$ 0.95, 2.35, 2.08, 8.18, 167.56, 10.85, 160.49, 47.93, 2.34, 3.38, 53.58, 0.00, 0.34, 0.22. The coefficient of variation of each evaluation indicator was calculated according to the formula (10), $\delta_{i}=$ 0.12, 0.31, 0.28, 0.05, 0.17, 0.14, 0.24, 0.25, 0.22, 0.16, 0.29, 0.11. The weight of each evaluation indicator was calculated according to the formula (11), $W_{i}=$ 0.04, 0.11, 0.10, 0.02, 0.06, 0.05, 0.09, 0.09, 0.08, 0.06, 0.10, 0.04, 0.08, 0.10. Finally, the corresponding values in Wi and $R_{\Delta}$ were substituted into the formula (13), and the closeness compound fuzzy matter-element $R_{H}$was calculated:

$$R_{H}=\left[ \begin{matrix} & M_{1} & M_{2} & M_{3} & M_{4} & M_{5} & M_{6} & M_{7} & M_{8} & M_{9} \\ & & & & & & & & & \\ H_{j} & 0.94 & 0.26 & 0.64 & 0.60 & 0.46 & 0.35 & 0.55 & 0.45 & 0.33 \end{matrix} \right]$$

The calculated closeness descreased in the order *M*_1_＞*M*_3_＞*M*_4_＞*M*_7_＞*M*_5_＞*M*_8_＞*M*_6_＞*M*_9_＞*M*_2_. In terms of difference, each corresponding dose groups between ARPCM and HRPCM were smaller, namely: $\left| M_{4}-M_{7} \right|=0.05, \left| M_{5}-M_{8} \right|=0.01, \left| M_{6}-M_{9} \right|=0.02$. Therefore, it can be determined that in high dose group of the two drugs, namely 1.89g/kg, the efficacy on regulating the immune function of HRPCM is stronger than ARPCM. In terms of the medium- and low- dose groups, the effect of HRPCM was slightly stronger than ARPCM.
